# Integrated kinetic, thermodynamic, and statistical investigation of aniline blue dye removal using magnesium silicate nanoparticles

**DOI:** 10.1038/s41598-025-18726-z

**Published:** 2025-09-24

**Authors:** Ahmed Magdy, Magdi E. A. Zaki, Maysa R. Mostafa, Gehad G. Mohamed, Sami A. Al-Hussain, Omar A. Fouad

**Affiliations:** 1 Chemistry Department, Faculty of Science, Cairo University, 12613 Cairo, Giza Egypt; 2 Department of Chemistry, Faculty of Science, Imam Mohammad Ibn Saud Islamic University (IMSIU), Riyadh, 11623 Saudi Arabia; 3 Nanoscience Department, Basic and Applied Sciences Institute, Egypt-Japan University of Science and Technology, New Borg El Arab, Alexandria, 21934 Egypt

**Keywords:** Aniline blue dye, Adsorption kinetics, X-ray diffraction, Transmission electron microscope, Magnesium silicate, Ceramic nanoparticles, Box-Behnken design, Environmental sciences, Inorganic chemistry, Materials chemistry

## Abstract

Magnesium silicate nanoparticles were found to be an effective adsorbent for aniline blue dye from wastewater. Using the sol-gel technique, magnesium silicate nanoparticles were synthesized and characterized using X-ray diffraction (XRD), transmission electron microscope (TEM), Brunauer-Emmett-Teller (BET) surface area, and fourier transform infrared spectroscopy (FTIR). Aniline blue removal was done at room temperature, pH 4, and a dosage of 3 g/L was about 99% in the first 30 min. Magnesium silicate can also be used for four cycles to adsorb Aniline blue dye without the need for disposal, which supports the principle of recycling. Response surface methodology was used for statistical analysis to investigate the impact of the factors. By studying the isotherms, kinetics, and thermodynamics, it became clear that the adsorption process involves a physical interaction that adheres to the Freundlich isotherm, follows pseudo-first-order kinetics, with the boundary layer (film) diffusion identified as the rate-determining step. The process is endothermic and spontaneous. Magnesium silicate nanoparticles were successfully used to remove dye contaminants from various actual water samples. The material’s reliability and potential for real-world environmental applications were demonstrated by the high efficiency and consistent adsorption results across multiple sample types.

**Supplementary Information:**

The online version contains supplementary material available at 10.1038/s41598-025-18726-z.

## Introduction

Water is considered an essential resource for survival. Growing demand for it makes recycling and treating water, especially with economic and eco-friendly methods, a critical issue for many years. Human activities, such as industrial and agricultural processes, contribute to water pollution. According to statistics, 2.4 billion people are vulnerable to various waterborne diseases, and about 1 billion lack adequate access to fresh water. Due to ongoing industrial development and population growth, the need for freshwater increases. The United Nations World Water Development Report states that by 2050, approximately 6 billion people could be affected by water access issues. Earth’s freshwater makes up only about 2.5%, even though 70% of its surface is covered with water. Changes in water characteristics caused by certain substances can turn water into wastewater. One major contaminant is organic dyes^1,2^.

Dye molecules consist of chromophores, which are responsible for their coloration, and auxochromes, which enhance the dye’s color. The colors produced by dyes depend on the wavelength of light absorbed by the chromophores and auxochromes^3,4^. In various industrial applications such as textiles, printing, paint, food, and cosmetics, dyes are commonly utilized as coloring agents^5^. Consequently, many coloring agents—particularly organic dyes—have entered industrial effluent^6^. The chemical structures of dyes and their stability vary in application and resistance to standard wastewater treatment^7^. Even though dyes are organic materials, their complex molecular structures make them resistant to biological degradation and durable in conventional wastewater treatment procedures^8^. Discharging inadequately treated industrial effluent into water sources may lead to pollution, and the existence of complex structure dye compounds in water environments has raised serious concerns for the health of humans^9^. Eliminating dyes like aniline blue (AB), which is an anionic dye with an acidic quality that pollutes water^10^. It is a triphenylmethane dye that has three sulfonic acid groups, making it acidic, C_32_H_25_N_3_O_9_S_3_Na_2_, 737.73 g/mol, and has maximum absorption at $\:{\lambda\:}_{max}$ = 597 nm. This dye is commonly employed in the textile industry to color a wide range of textiles. It is considered xenobiotic because of its distinct chemical structure, which makes it difficult to break down. Numerous aquatic species, including plants, animals, and microbes, are poisoned by it. Because aniline blue is released into wastewater that has not been adequately treated, it contaminates water. Adsorption is a highly efficient method for eliminating aniline blue from wastewater^11^.

Several chemical and physical techniques were investigated to eliminate synthetic organic dyes from water, including adsorption, advanced oxidation process, membrane separation, electrolysis, chemical precipitation, and photocatalytic degradation. While biological processes and advanced oxidation processes are excellent chemical-free methods, they have drawbacks in the form of by-products and restricted scalability. Chemical precipitation has a low operating cost, but some drawbacks include using chemicals, creating secondary pollutants, and insufficient removal of dyes. With very little chemical consumption, photocatalytic degradation also efficiently eliminates the contaminants; nevertheless, some drawbacks include restricted light penetration, slower reaction kinetics, and the generation of by-products^12^. Conversely, the adsorption process is frequently considered the most highly effective and economically-friendly approach for water treatment owing to its simplicity, green method, and successful removal of organic dyes and inorganic pollutants^13^. While there are some other materials such as conjugated polymers^14^, biomass-based materials^15^, composite materials^16^, and chemically modified adsorbents^17^ that also have efficiency in the wastewater treatment process, the use of nanoparticles is one of the best of those materials used because of their small volume, large surface area, vacant adsorbate sites, and high porosity^18^. Mesoporous^19^, carbon nanotube^20^, magnetic^21^, and nanoceramic materials^22^ are examples of nanomaterials that are frequently employed as innovative adsorbents. The term “nanoceramic” refers to a wide range of ceramic materials with at least one component whose dimensions are within the nanometer range (1–100 nm). These materials include fibers, tubes, sheets, rods, thin films, and nanostructured particles developed for various sectors and applications. Because ceramic materials, such as magnesium silicate, offer high surface areas and high porosity in addition to their mechanical and chemical resistance, than polymer-based adsorbents, such as alumina, silica, zirconia, or calcium phosphate, they are frequently utilized as adsorbents in the removal of environmental contaminants^23^. To achieve the required properties of nanoparticles, it is essential to choose an appropriate preparation method and meticulously regulate its influencing components^24,25^.

Y. Guan et al. have used acidified polygorskite/BiOI composites in the removal of aniline blue dye by the adsorption-photoactivity method. Despite the initial concentration of dye has reached 50 mg/l, the removal percentage was 90%, and the contact time was 360 min, it’s time-consuming^26^.

Liu Q et al. used chitosan-coated activated carbon for the removal of aniline blue from wastewater using the adsorption method. Even though this adsorbent has a high surface area and porosity, the removal rate reached 80% in 100 min, which is considered a shortcoming in the removal process^27^.

Named for the German naturalist Johann Forster, Mg_2_SiO_4_ is the chemical formula for the crystalline magnesium silicate known as magnesium silicate. It belongs to the olivine group^28^. This material is classified as a nanoceramic due to its remarkable physical properties^29^. They have excellent chemical stability, a low dielectric value, a relatively small thermal expansion coefficient, outstanding thermal insulation, and high refractoriness (≥ 1890 °C). Magnesium silicate nanoparticles are extensively utilized in the manufacturing of diverse technological components, such as dielectric substrates, biomaterials, and solid oxide fuel cells^30–32^.

Therefore, this study aims to overcome the limitations reported in previous studies, such as moderate removal efficiency, long contact times, and low initial concentration, by employing magnesium silicate nanoparticles as an efficient and rapid adsorbent for aniline blue dye. The proposed material is expected to exhibit a higher removal percentage within a shorter equilibrium time, providing a promising alternative for effective wastewater treatment.

## Experimental

### Chemicals and materials

All chemicals, materials, and devices that are used in this work are listed in the supplementary file.

### Preparation of magnesium silicate nanoparticles

**Fig. 1:**
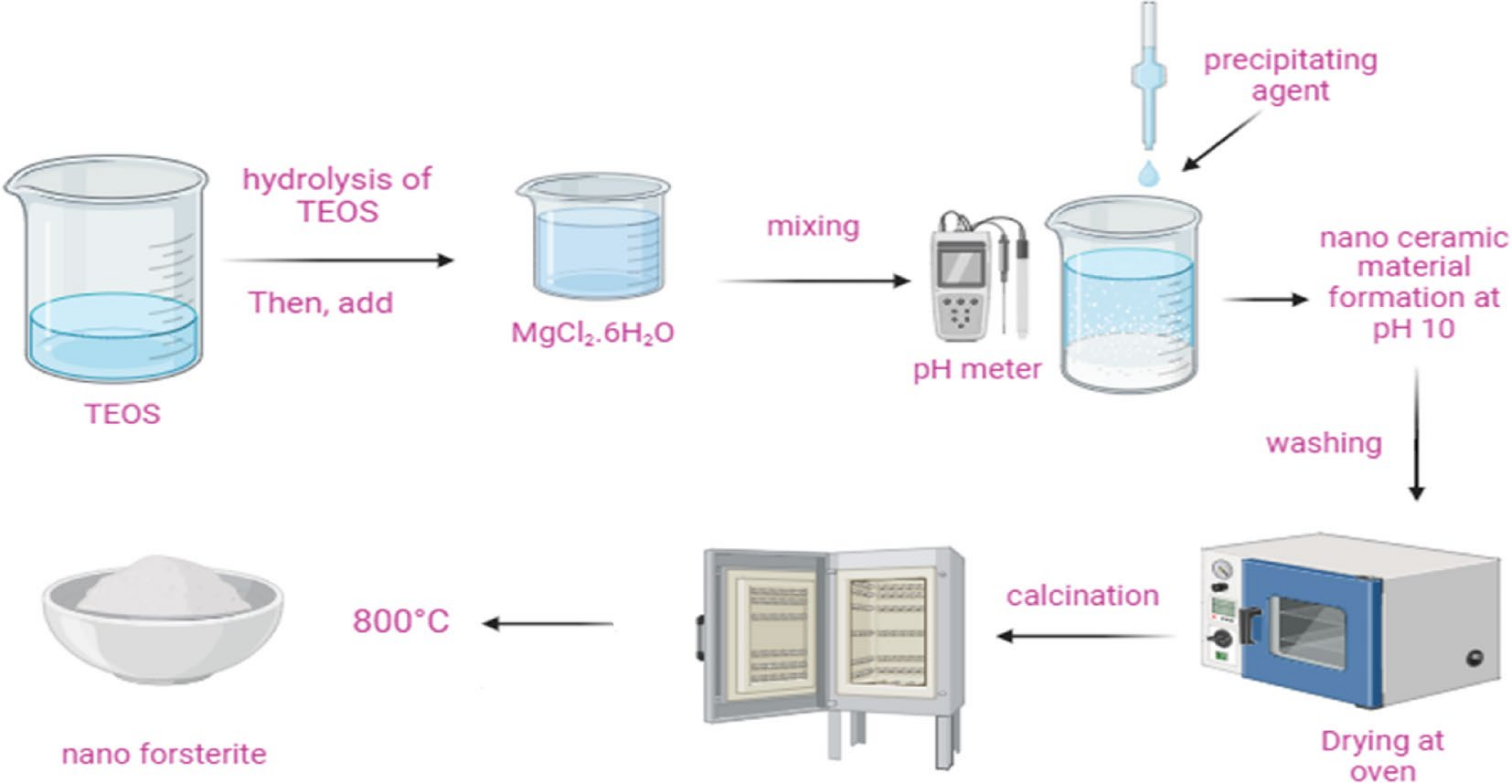
Preparation method of magnesium silicate nanoparticles.

As shown in Fig. 1, magnesium silicate was synthesized utilizing the sol-gel method. The initial precursors for magnesium and silicon consisted of MgCl_2_.6H_2_O and tetraethyl orthosilicate (TEOS). TEOS underwent hydrolysis using a combination of 250 mL of distilled water and 50 mL of ethanol (EtOH), with stirring conducted for 2 h at a temperature of 80 $\:^\circ\:C$. Following this, a solution comprising 1 mol of MgCl_2_ was added and stirred for 30 min under identical conditions. The solution’s pH was adjusted to 10 through the addition of NH_4_OH. Subsequently, the solution remained undisturbed for 24 h, resulting in a highly viscous solution. The viscous solution underwent filtration, followed by the drying of the precipitate, which was subsequently calcined at a temperature of 800 $\:^\circ\:C$.

### Nanoparticles characterization

A German-based Bruker D8 Discover diffractometer assessed the material’s crystallinity. Measurements were conducted at forty kV and a current of forty mA within the 3º ≤ 2θ ≤ 80º range. The internal features were analyzed using a JEOL-JEM 2100 transmission electron microscope (TEM) from Japan. The specific surface area of magnesium silicate nanoparticles was determined using a Quanta Chrome automated gas sorption apparatus. The contact angle is determined by using a goniometer.

### Point of zero charge

The salt addition method was used to identify the surface charge polarity of the substance and provide insights into its surface characteristics. Each beaker in the series held 50 ml of 0.01 N NaCl solution. The pH of the initial solution was modified from 2 to 11, utilizing 0.01 N HCl and 0.01 N NaOH. Subsequently, 0.1 g of nanoparticle was introduced to each beaker and maintained on the stirrer for 24 h to achieve equilibrium. Subsequently, the ultimate pH was determined. The zero-point charge was derived by graphing the initial pH (pH_i_) against the change in pH (∆pH = pH_f_ - pH_i_). The pH_pzc_, or point of zero charge, refers to the pH at which the initial and final pH are equivalent. Prior to this point, the surface carries a positive charge because of protonation, whereas after this point, it carries a negative charge.

### Investigation of adsorption

**Fig. 2:**
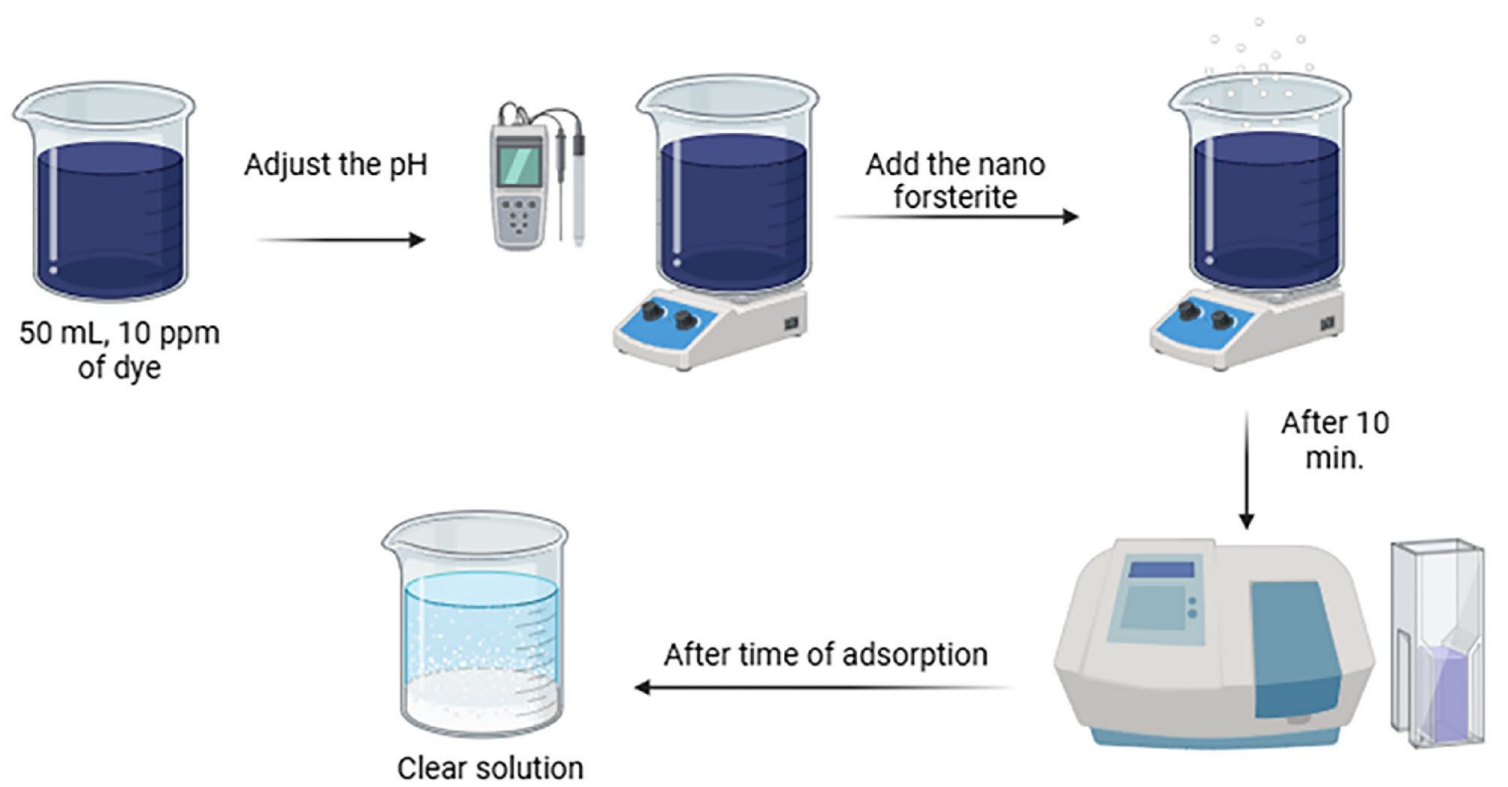
Steps of dye removal by the adsorption process.

Figure 2 presents a schematic diagram illustrating the steps of the adsorption process utilized for dye removal. The adsorption studies were conducted at a constant temperature of (25.0 ± 1.0 °C), using 0.15 g of adsorbent and a 50 ml dye solution. NaOH or HCl with a concentration of 0.1 M has been used to adjust the dye solutions’ pH. The experiments were carried out by stirring the solution of dye and nanoparticles at a consistent rate in a 250-ml conical flask. Following the required time, the withdrawal of the dye from the solution was accomplished by utilizing a 45 μm syringe filter. Many factors were examined, including pH value, contact duration, shaking speed, adsorbent dose, and starting dye concentration.

The equation presented as (1) was utilized to determine the percentage of dye removal:

1 $$\:\text{\%}\:\text{R}\text{e}\text{m}\text{o}\text{v}\text{a}\text{l}=\frac{\text{C}\text{o}-\text{C}\text{e}}{\text{C}\text{o}}\:\times\:100$$

The variable Co represents the initial concentration of dye measured in (mg/L), whereas C_e_ represents the dye concentration after adsorption in the same unit. Moreover, the adsorption capacity of the adsorbent, q (measured in milligrams of dye per gram of dry adsorbent), could be determined using Eq. (2):

2 $$\:\text{q}=\frac{\left(\text{C}\text{o}\:-\text{C}\text{e}\right)\times\:\text{V}\:}{W}$$

V represents the solution’s volume in liters, whereas W represents the mass of the dry adsorbent in grams.

### Data analysis

The study used the Behnken box design (BBD), a kind of surface response technique utilized for process optimization in the industrial sector. In contrast to traditional experimental designs that mainly assess average factor effects, the BBD facilitates the achievement of optimum outcomes with fewer experimental runs, thereby reducing expenses^33^. The BBD is especially adept at comprehensively investigating the design space to ascertain the optimal combinations of parameters that either enhance or diminish the response^34^. Following the acquisition of experimental data, a model was performed using a polynomial equation. Analysis of variance (ANOVA) was used to identify the factors and interactions influencing the elimination percentage. A residual analysis was performed to assess the model’s validity by comparing predicted results with actual outcomes, thereby evaluating the model’s robustness and identifying any deviations from its predictions. Contour plots were then generated to illustrate the synergistic impacts of three distinct variables (pH, dye concentration, and dose) on the elimination process. Different plots elucidate the interrelationships among different parameters throughout the elimination process. An optimization procedure is conducted using the model to ascertain the ideal circumstances that enhance the removal percentage. All analyses were performed with Design Expert software version 13.

## Results and discussion

### Magnesium silicate nanoparticles characterization

#### XRD

**Fig. 3:**
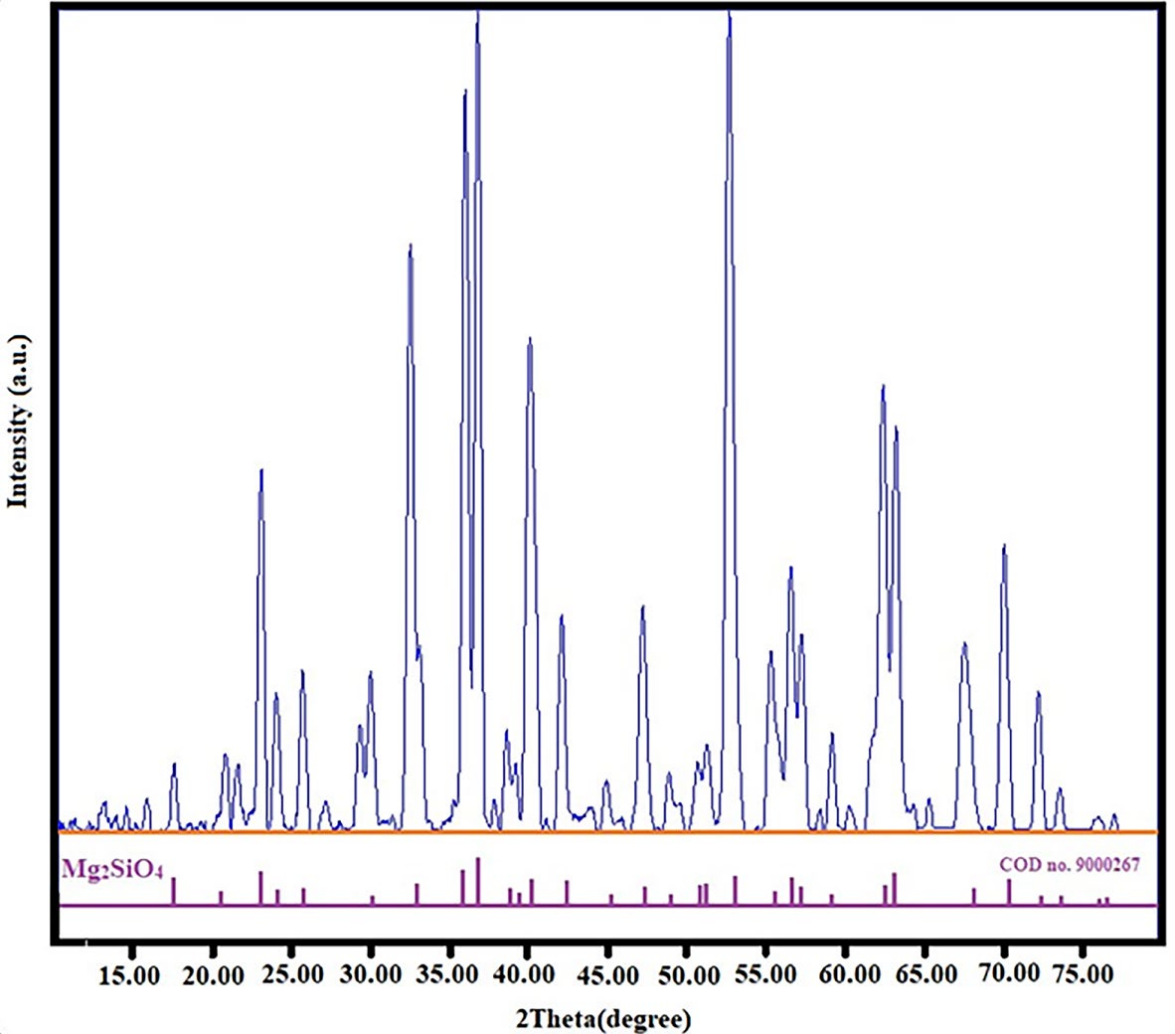
XRD pattern of magnesium silicate nanoparticles.

Figure 3 presents the X-ray diffraction (XRD) pattern of magnesium silicate nanoparticles. The XRD pattern of the sample displayed distinct peaks at various angles, specifically at 17.56°, 20.77°, 23.04°, 24.02°, 25.66°, 29.95°, 32.47°, 35.91°, 38.53°, 40.00°, 47.09°, 50.58°, 51.13°, 52.55°, 55.19°, 56.41°, 63.05°, 64.12°, 67.39°, 73.37°, and 76.84°. The specified angles relate to unique crystal planes, including [020], [110], [021], [101], [111], [121], [130], [131], [041], [140], [042], [103], [151], [222], [241], [061], [321], [223], [170], [134], and [270]. The crystal planes are connected to magnesium silicate. The data aligned with the standard COD no. 9,000,267 and exhibited an orthorhombic structure defined by the P b n m (62) space group. The Scherrer equation was applied to establish the crystallite size, resulting in an average size of 16.81 nm^35–37.^ Consequently, the XRD validated the magnesium silicate phase in the established sample. This sample underwent a thorough examination utilizing a different methodology and was later employed as an absorbent to remove Aniline blue.

#### Transmission electron microscope (TEM)

**Fig. 4:**
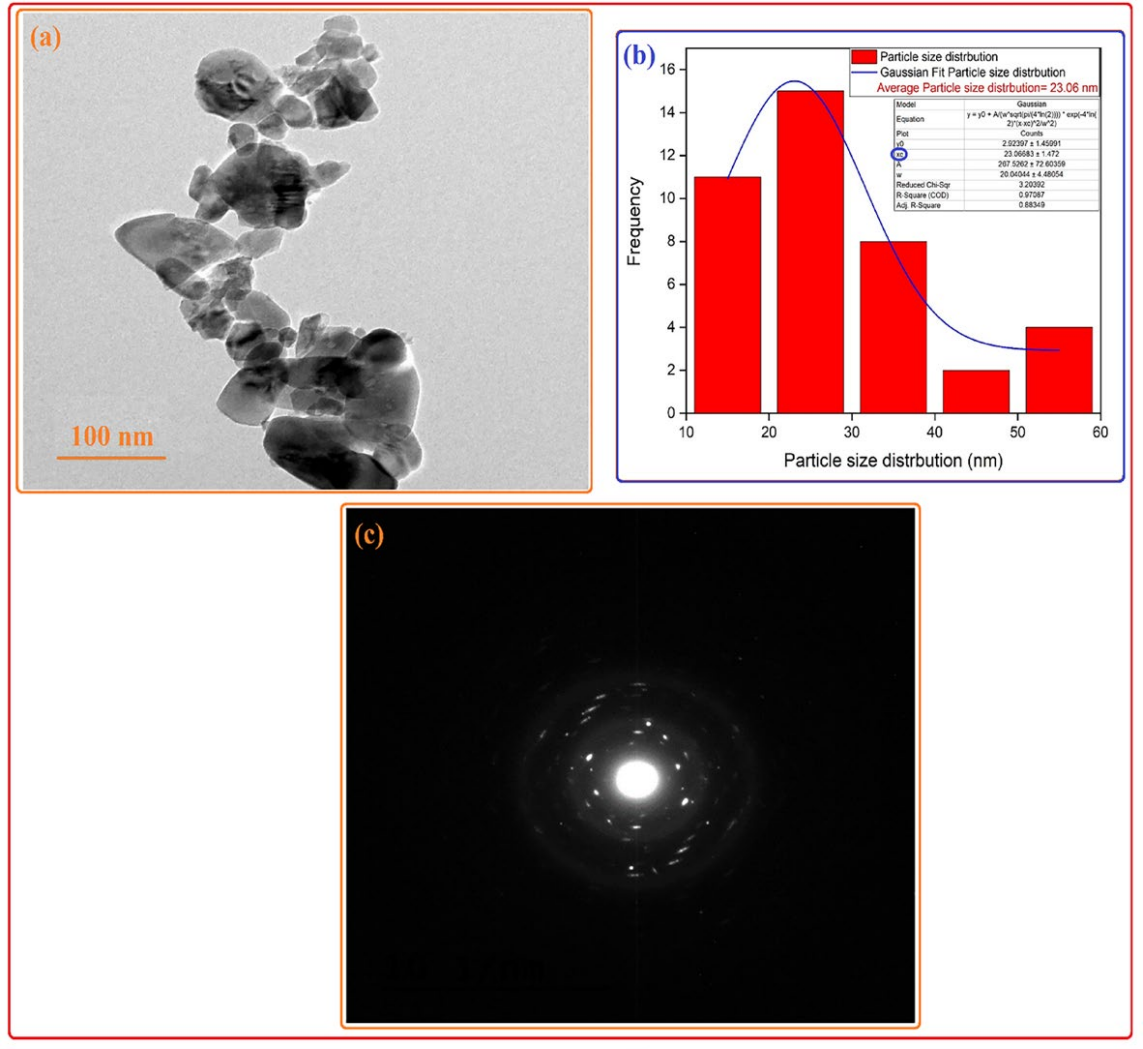
(**A**, **B**, and **C**). High-resolution transmission electron microscopy (**A**), distribution of particle size (**B**), and selected area electron microscope (SAED) images (**C**) for magnesium silicate nanoparticles.

Figure 4A presents the analysis of the synthesized nano-magnesium silicate through TEM imaging. The transmission electron microscopy (TEM) examination indicated that magnesium silicate nanoparticles exhibit irregular and spherical shapes, demonstrating homogeneity and crystallinity. The elevated crystallinity was further validated through the analysis of the selected area electron diffraction (SAED) image (Fig. 4C), which displayed a distinct and orderly configuration of lattice edges^38^ The particle size of the nano magnesium silicate was determined through a Gaussian simulation and a histogram constructed by employing Java 1.8.0 172 with the ImageJ (1.53e) application^24^. As shown in Fig. 4B, the sizes ranged from 11.3 to 54.9 nm, with an average particle size of 23.06 nm. Consequently, the TEM data were consistent with the XRD data and Scherrer equation computation since it validates the elevated crystallinity and nano-sized of the synthesized magnesium silicate sample.

#### Brunauer-Emmett-Teller (BET)

**Fig. 5:**
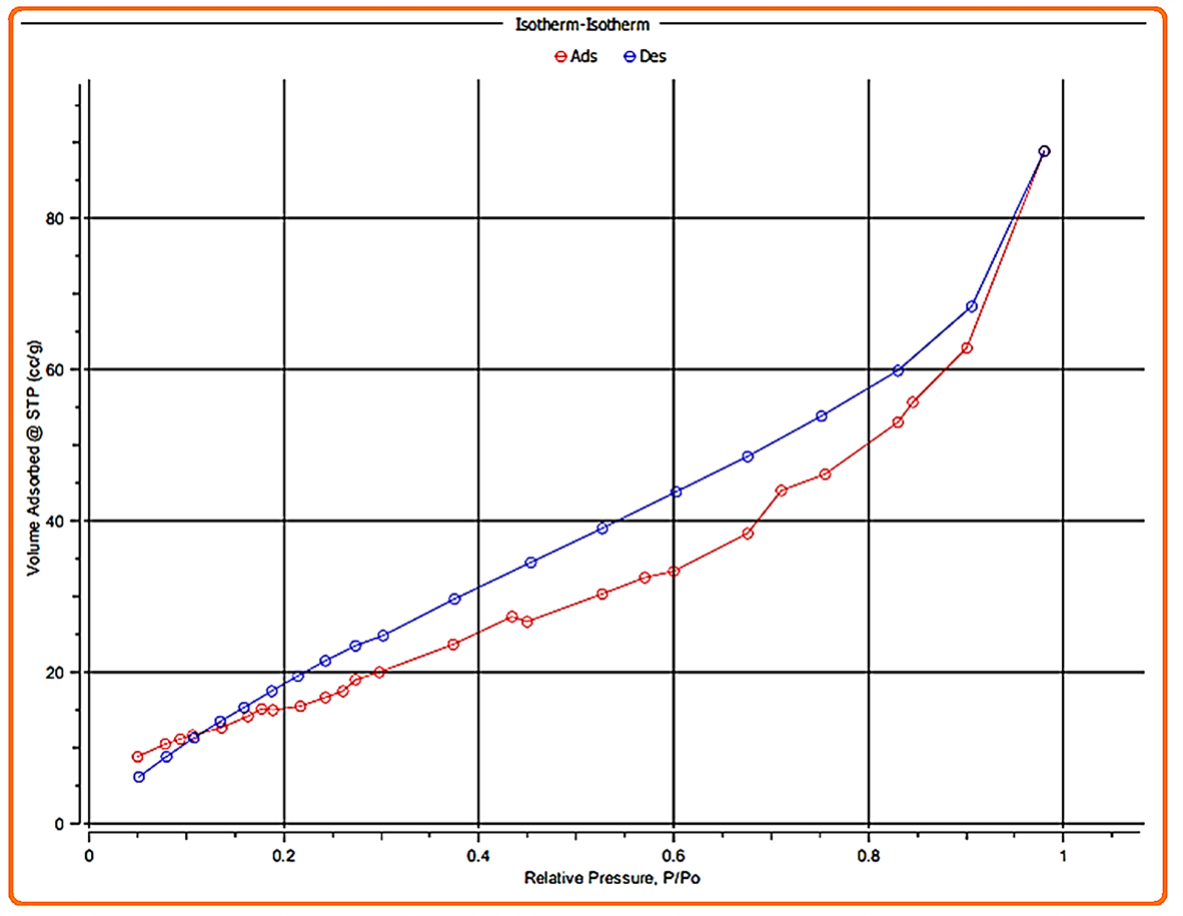
Nitrogen adsorption-desorption isotherms for magnesium silicate nanoparticles.

The nano magnesium silicate’s specific pore size, pore volume, and surface area were assessed by applying the nitrogen adsorption-desorption isotherm. Figure 5 reveals an isotherm hysteresis loop of nano magnesium silicate, characterized as a type IV-(a)-H3 loop, where an elevated volume adsorbed is observed at an associated pressure of 0.9^39^. The BET study demonstrated that the nano magnesium silicate exhibited an average pore size of 5.35 nm, a pore volume of 0.245 cm^3^/g, and a surface area of 90.75 m^2^/g. The results demonstrate that the nano magnesium silicate possesses considerable surface area and a mesoporous character^40^.

#### Fourier transform infrared spectroscopy (FTIR)

**Fig. 6:**
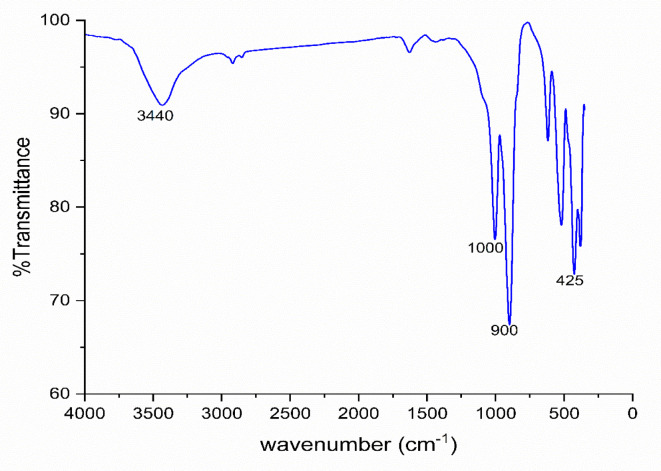
FTIR of magnesium silicate nanoparticles.

As shown in Fig. 6, the FTIR spectrum of the synthesized magnesium silicate nanoparticles shows a strong absorption band in the region around 1000 cm⁻¹, which is characteristic of the asymmetric stretching of Si–O–Si bonds, confirming the formation of a silicate network^41^. Additionally, an absorption near 900 cm⁻¹ due to Si–O–Mg interactions^41,42^. Bands observed in the lower wavenumber region (around 450 cm⁻¹) are attributed to Mg–O and Si–O bending vibrations, supporting the successful incorporation of magnesium into the silicate framework^42^. A broad band around 3400 cm⁻¹, which is attributed to O–H, due to some adsorbed water on the surface of magnesium silicate^41^.

Following the analysis of magnesium silicate nanoparticles, a comparison was made with other previously studied nanomaterials that exhibit similar characteristics, including mesoporous structures^43^ and high surface areas^44^. Recent findings have shown that nano magnesium silicate can be an adsorbent for removing dyes, such as the Aniline blue dye.

### Study the variables influencing the adsorption process

The investigation focused on various factors affecting dye removal from aqueous solutions, including stirring rate, initial dye concentration, pH, contact duration, and adsorbent dosage, to determine the optimal parameters that exhibit the highest removal efficiency for the dye studied.

#### Effect of pH and point of zero charge

**Fig. 7:**
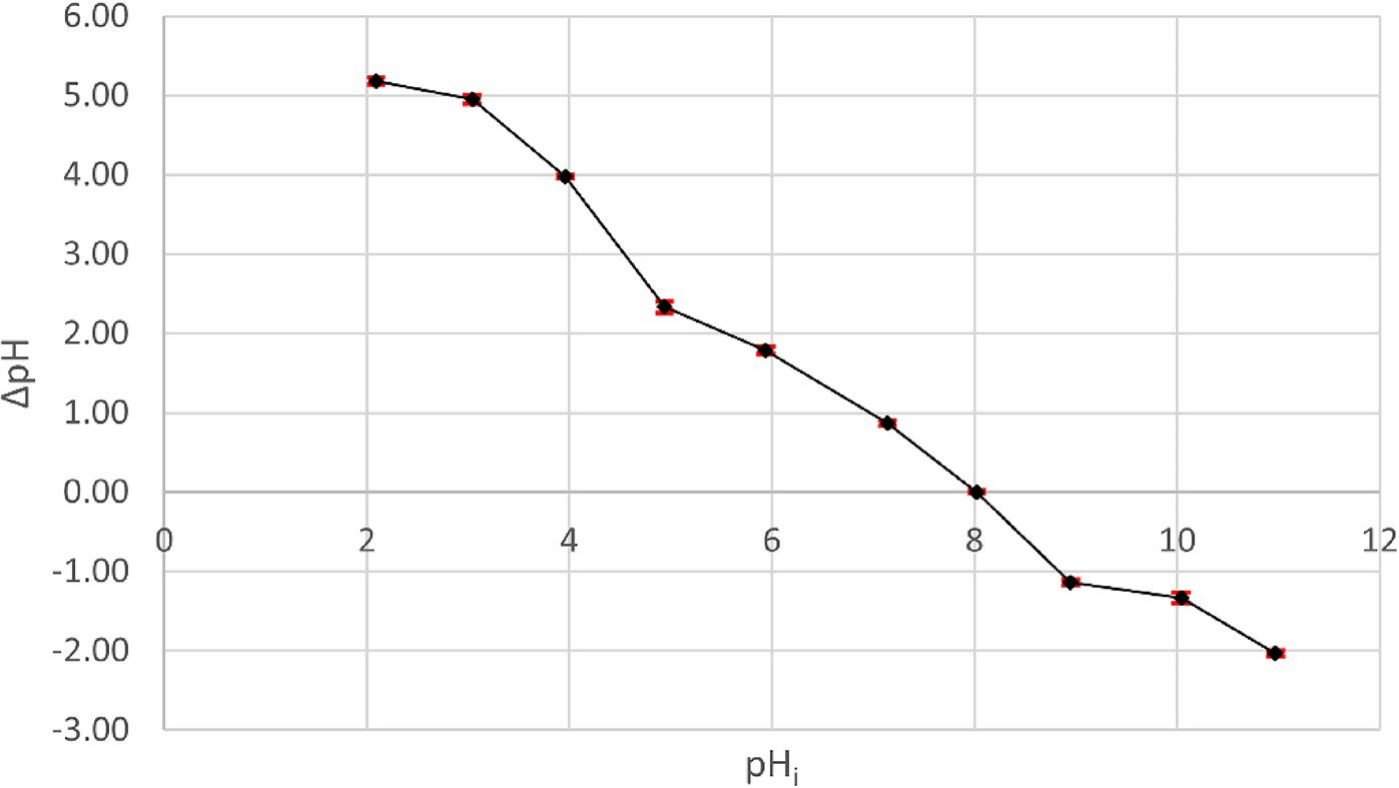
Zero Point Charge measurement of magnesium silicate nanoparticles.

**Fig. 8:**
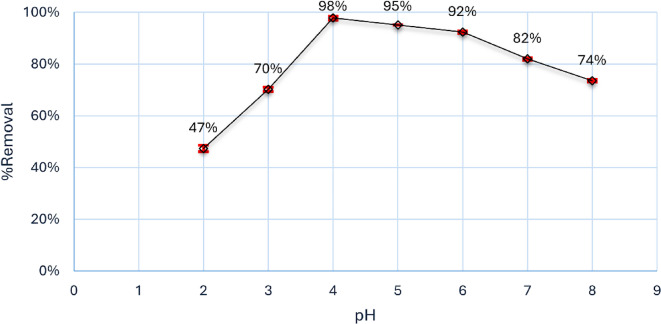
Effect of pH on adsorption of Aniline blue dye onto magnesium silicate nanoparticles.

The pH of the solution is an essential factor that impacts the entire adsorption process due to the charge on the outer layer of the adsorbent can be changed by it. Through participating in that operation, the interaction between the dye and the adsorbent nanoparticles will alternate between attracting and repelling each other^45^. The adsorption capacity is pH-dependent and will change based on the kind of dye (if it has a positive or negative charge) and its interaction with magnesium silicate. As shown in Fig. 7, the pH_pzc_ of magnesium silicate nanoparticles was 8, indicating that below 8, the surface of magnesium silicate has a positive charge, and after that, the charge was negative^46^. The adsorption process increases in an acid medium because of the positive charge of magnesium silicate in this medium and the presence of Aniline blue dye, an anionic dye containing a sulphonate group, leading to an electrostatic attraction between the magnesium silicate nanoparticles and the aniline dye. As shown in Fig. 8, by using 0.10 g of magnesium silicate/50 ml of 10 mg/l of Aniline blue dye at pH 4, the % removal was about 98% after 30 min. Nevertheless, it gradually decreased until it reached a value of 59% at pH 9, primarily because of the deprotonation of the sulphonate group^45^. Consequently, the anionic magnesium silicate nanoparticle counteracted the dye’s total negative charge.

#### Effect of adsorbent dosage

**Fig. 9:**
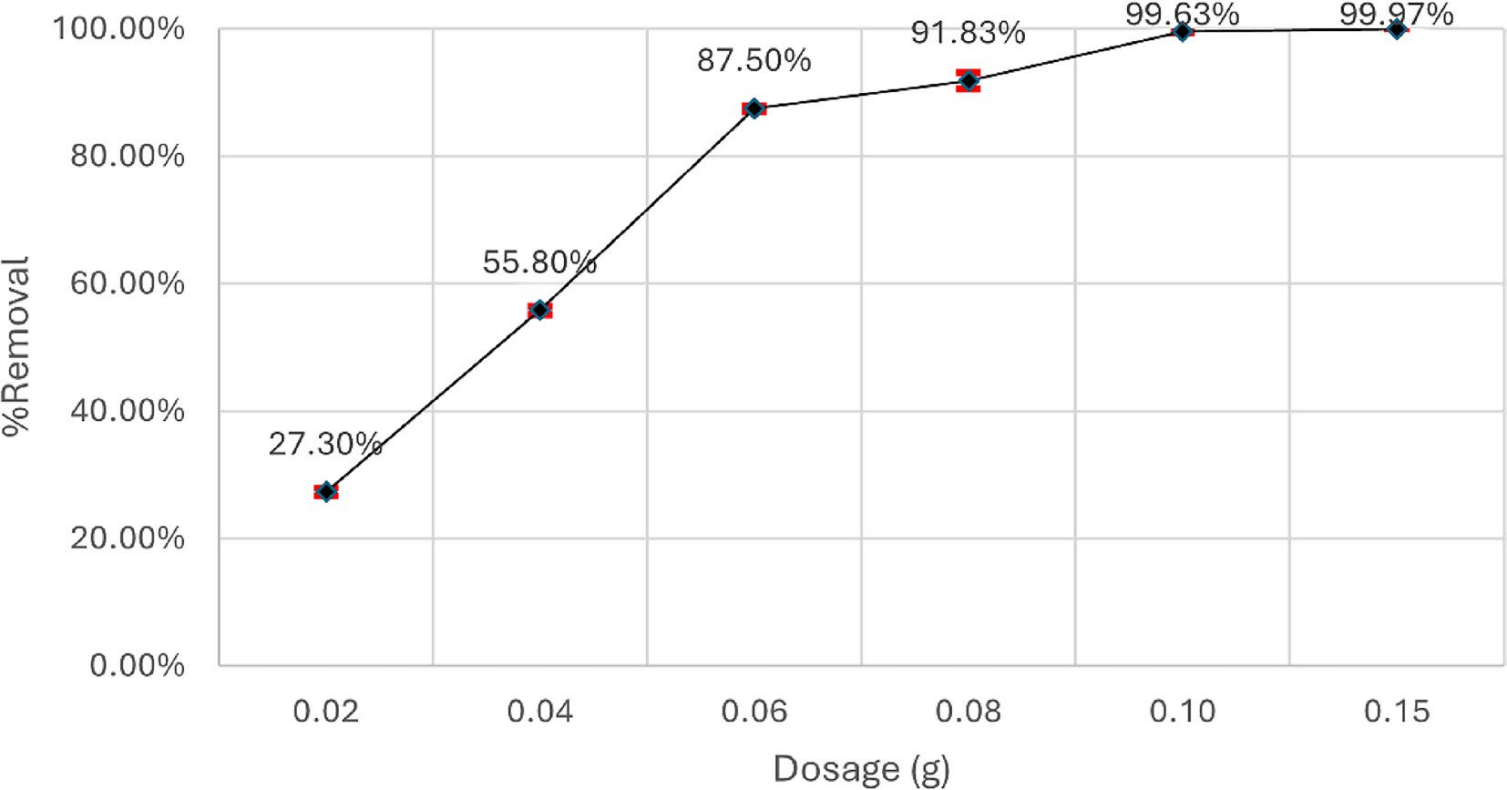
Impact of nanoparticles dosage on the adsorption of Aniline blue dye onto magnesium nanoparticles.

The investigation focused on the adsorption of aniline blue dye in an aqueous solution utilizing magnesium silicate nanoparticles. Figure 9 shows that the removal percentage increased from 27.3 to 99.97% as the dosage of nanoparticles was raised from 0.02 g to 0.15 g at pH 4 while maintaining the remaining parameters constant, followed by absorbance measurement using a UV spectrophotometer. The enhanced adsorption is because of the expanded surface area and active sites of nanoparticles^47^. It is also noted that more than 90% removal can be reached with 0.08 g.

#### Effect of initial dye concentration

**Fig. 10:**
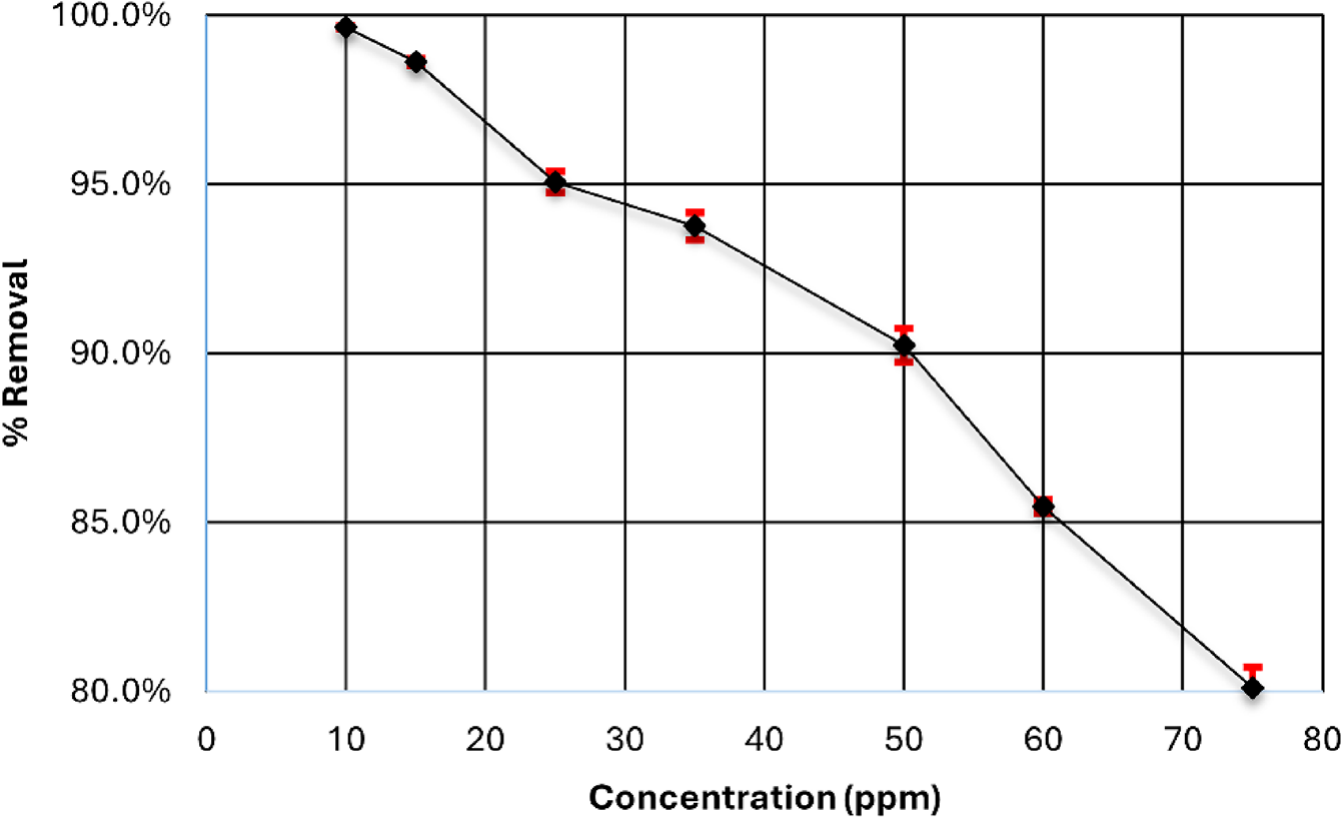
Effect of initial dye concentration on the adsorption of the Aniline blue dye.

Initial dye concentration has a significant effect on the removal of dye by the adsorption process. The impact of this parameter relies on the direct relationship between dye concentration and adsorbent surface site availability^48^. Usually, as the initial dye concentration rises, the dye removal efficiency decreases, probably because there are too many adsorption locations on the adsorbent’s outer layer. On the other hand, the high dye concentration leads to a higher adsorbent capacity because a stronger force drives the transfer of mass at higher dye concentrations^49^. By analyzing the impact of the initial dye concentration at pH 4 and using 0.15 g of adsorbent at ambient temperature with changing the concentration from 10 to 75 ppm while the remaining parameters were constant, it was observed that the nanoparticles have the ability to remove dye with more than 80% to 75 ppm of aniline blue, as shown in Fig. 10. The %Removal of dye decreased when the initial concentration of dye increased due to the pores of active sites of nanoparticles being filled with dye^50^.

#### Effect of contact time

**Fig. 11:**
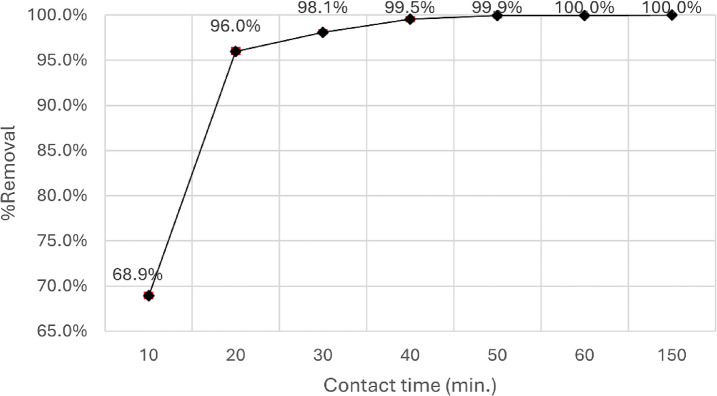
Impact of contact time on the adsorption of Aniline blue dye.

Equilibrium time is another crucial factor in a highly effective technique for treating wastewater. With increasing contact duration of adsorbent with adsorbate the removal tendency increases but at certain point it becomes constant. For optimal efficiency in the adsorption dye removal process, the materials must exhibit a rapid removal rate and possess a substantial capacity. As shown in Fig. 11, the impact of contact time is studied at pH 4 by using 0.15 g of nanoparticles, 10 mg/l, and 50 ml of dye at ambient temperature, the % of removal rapidly increased, reaching 98% in the first 30 min.

#### Acceleration speed’s effect

**Fig. 12:**
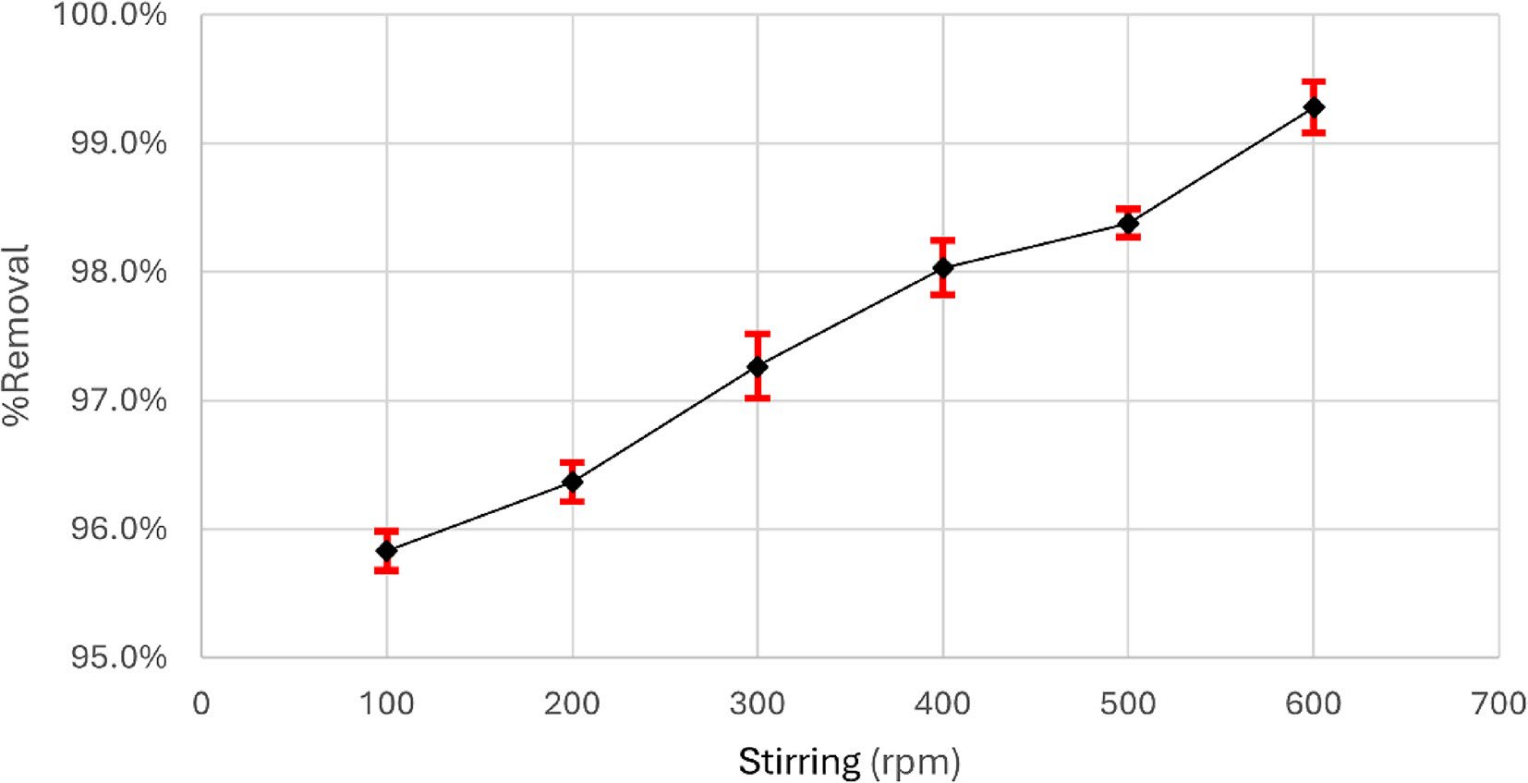
Impact of agitation speed on the adsorption of Aniline blue dye.

It affects how the solute diffuses through the solution and how the outer boundary layer is formed^51,52^. The impact of the stirring rate was investigated by altering it from 100 to 600 rpm while maintaining the other parameters constant. Figure 12 shows that the elimination percentage is not significantly influenced by changing the stirring rate and making the other parameters constant; the removal is still more than 97%, even at any rpm.

### Mechanism of adsorption

**Fig. 13:**
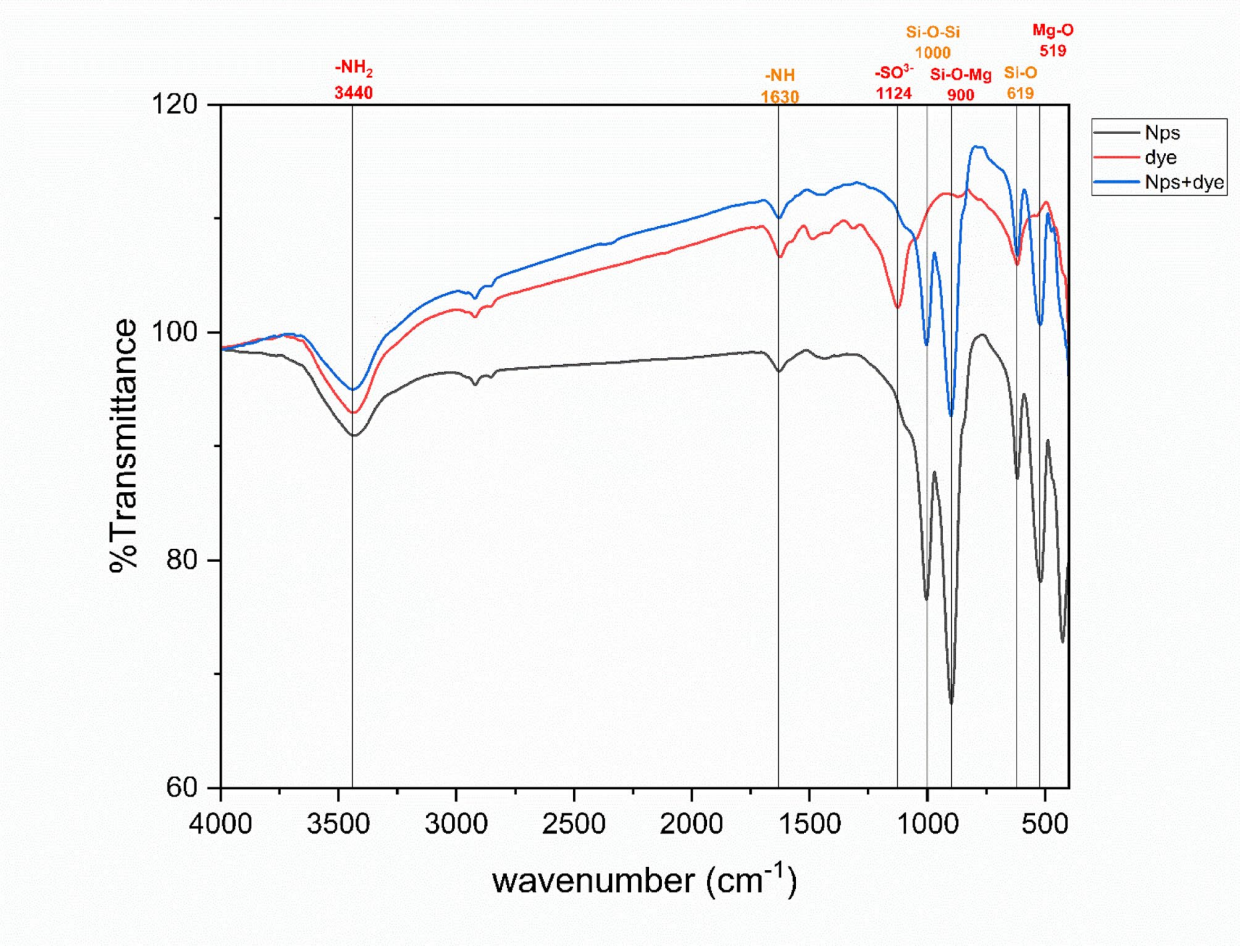
FTIR of nanoparticles, dye, and nanoparticles with dye.

The adsorption mechanism of aniline blue dye onto the synthesized magnesium silicate nanoparticles was investigated by comparing the FTIR spectra of the dye, the nanoparticles, and the composite after adsorption, as shown in Fig. 13. The FTIR spectrum of pure aniline blue displayed characteristic peaks assigned to the sulfonate groups (–SO₃⁻) around 1120–1200 cm⁻¹, aromatic C = C stretching in the region 1600–1500 cm⁻¹, (–NH) stretching band at 1625 cm^− 1^, and characteristic peak attributed to amine group (–NH_2_) around 3400 cm^− 1^^53^. The spectrum of magnesium silicate nanoparticles exhibited distinct peaks near 1000–1100 cm⁻¹, corresponding to Si–O–Si and Si–O stretching vibrations, along with minor bands associated with O–H bending and stretching from surface hydroxyl groups. After adsorption, the FTIR spectrum of the NPs with dye composite showed significant modifications: The Si–O stretching band was reduced in intensity, indicating an interaction with dye molecules. New bands appeared in the 1120–1200 cm⁻¹ region, confirming the presence of SO₃⁻ groups from the dye, supporting successful adsorption. Additionally, there were minor shifts or changes in intensity in N–H band, indicating possible additional interactions, such as hydrogen bonding.

The Zeta potential analysis also revealed that the isoelectric point (IEP) of the nanoparticles is approximately pH 8, indicating that the surface is positively charged at acidic pH levels. Since the optimum pH for adsorption was pH 4, the nanoparticles carried a significant positive surface charge, enabling strong electrostatic attraction with the negatively charged dye molecules. Therefore, the adsorption mechanism is primarily driven by electrostatic interactions, particularly between the protonated surface of the nanoparticles and the anionic sulfonate groups of the dye. The FTIR results also imply additional contributions from hydrogen bonding and van der Waals.

### Statistical analysis by using response surface methodology

Response Surface Methodology (RSM) was used to conduct a statistical study of the removal process to assess the influence of various elements on the experiment from a statistical perspective. The examination of regression coefficients and equations in the experimental design of RSM was conducted using Design-Expert 13 software. The software analyzes the impact of many parameters on the removal process and optimizes the experimental circumstances to get the maximum design response rate.

#### Box-Behnken design

Numerous designs, including BBD, three-level factorial design, and central composite design, may be used for input experimental data inside RSM modelling. The Box-Behnken design enables the development of a response surface model that delineates the interaction between the components and the response variable. This information is essential for understanding and enhancing the process’s performance^54^. This work used BBD design inside RSM to optimize the interplay of three parameters influencing the adsorption process: (A) pH, (B) concentration, and (C) dose. This design has 17 distinct experiments derived from alterations of the three preceding parameters. The BBD-RSM design was assessed to identify the most suitable models, including linear, quadratic, two-factor interaction (2FI), and cubic. The quadratic model was determined to be the best ideal for the design based on the sum of squares, F-value, and probability > F-value. A second-order polynomial equation (Eq. 3) was formulated from this model, illustrating the influence of the components on the removal ratio and their interactions.

3 $$\begin{gathered} \% Removal = \: - 94.56 + 40.39*A + 0.54*B + 1317.79*C - 0.0056*A*B + 7.4*A*C \hfill \\ \quad \quad \quad \quad \quad \quad \quad - 1.61*B*C - 3.74A^{2} - 0.0066B^{2} - 5189.5C^{2} \hfill \\ \end{gathered}$$

Where A, B, and C are symbols which identify pH, concentration, and dosage, respectively, AC, BC, and AB denote the coefficients of the mutual interactions, whereas A^2^, B^2^, and C^2^ represent the coefficients of the quadratic terms. The positive sign indicates the synergistic effect of interacting factors, whilst the negative sign delineates the competing influence of factors on dye removal^55^. Table 1 presents the values of three distinct factors across 17 experiments, alongside the actual and predicted removal percentage values.

**Table 1:** Statistical design matrix under BBD-RSM.

Run Order	Factor A: pH	Factor B: Conc. (ppm)	Factor C: dosage (g)	Actual Value of %removal	Predicted Value of %removal
1	4	10	0.02	34.2	36.27
2	4	75	0.15	79.43	76.31
3	4	75	0.02	28.91	31.54
4	8	75	0.08	57.93	56.78
5	4	10	0.15	96.23	94.65
6	4	50	0.08	83.04	84.82
7	8	50	0.15	72.82	75.37
8	2	50	0.02	8.57	4.29
9	8	50	0.02	21.93	21.51
10	4	50	0.08	85.04	84.82
11	8	10	0.08	70.23	69.25
12	4	50	0.08	85.53	84.82
13	2	10	0.08	47.53	48.02
14	4	50	0.08	85.3	84.82
15	2	50	0.15	50.23	52.38
16	4	50	0.08	85.2	84.82
17	2	75	0.08	36.1	37.74

#### Analysis of ANOVA outcomes and model fitting

ANOVA offers a statistical framework to assess the appropriateness of a model via comprehensive regression coefficient analysis. The tests include the F-test and the p-test to determine the model’s producibility and appropriateness. Table 2 illustrates the ANOVA findings from the BBD-RSM design, with a model F-value of 144.01 and the corresponding prob > F-value. The model is significant with a p-value of < 0.01%, suggesting a strong match. The F-value and p-value findings indicate an extremely low probability that the model’s F-value is due to random noise. Since the p-value must be less than 0.05 for the model terms to be considered significant^54^, A, B, C, A^2^, B^2^, and C^2^ are significant factors, but the other terms over 0.1 are not significant.

**Table 2:** ANOVA findings under BBD-RSM.

Source	Sum of Squares	df	Mean Square	F-value	*p*-value	
Model	11530.7	9	1281.19	144.01	< 0.0001	significant
A-pH	806.57	1	806.57	90.66	< 0.0001	
B-concentration	268.49	1	268.49	30.18	0.0009	
C-dosage	5106.47	1	5106.47	574	< 0.0001	
AB	1.29	1	1.29	0.1445	0.7151	
AC	8.8	1	8.8	0.9894	0.353	
BC	47.55	1	47.55	5.35	0.054	
A²	3559.08	1	3559.08	400.06	< 0.0001	
B²	177.73	1	177.73	19.98	0.0029	
C²	1991.19	1	1991.19	223.82	< 0.0001	
Residual	62.27	7	8.9			
Lack of Fit	58.18	3	19.39	18.94	0.0079	significant
Pure Error	4.1	4	1.02			
Cor Total	11592.97	16				

**Fig. 14:**
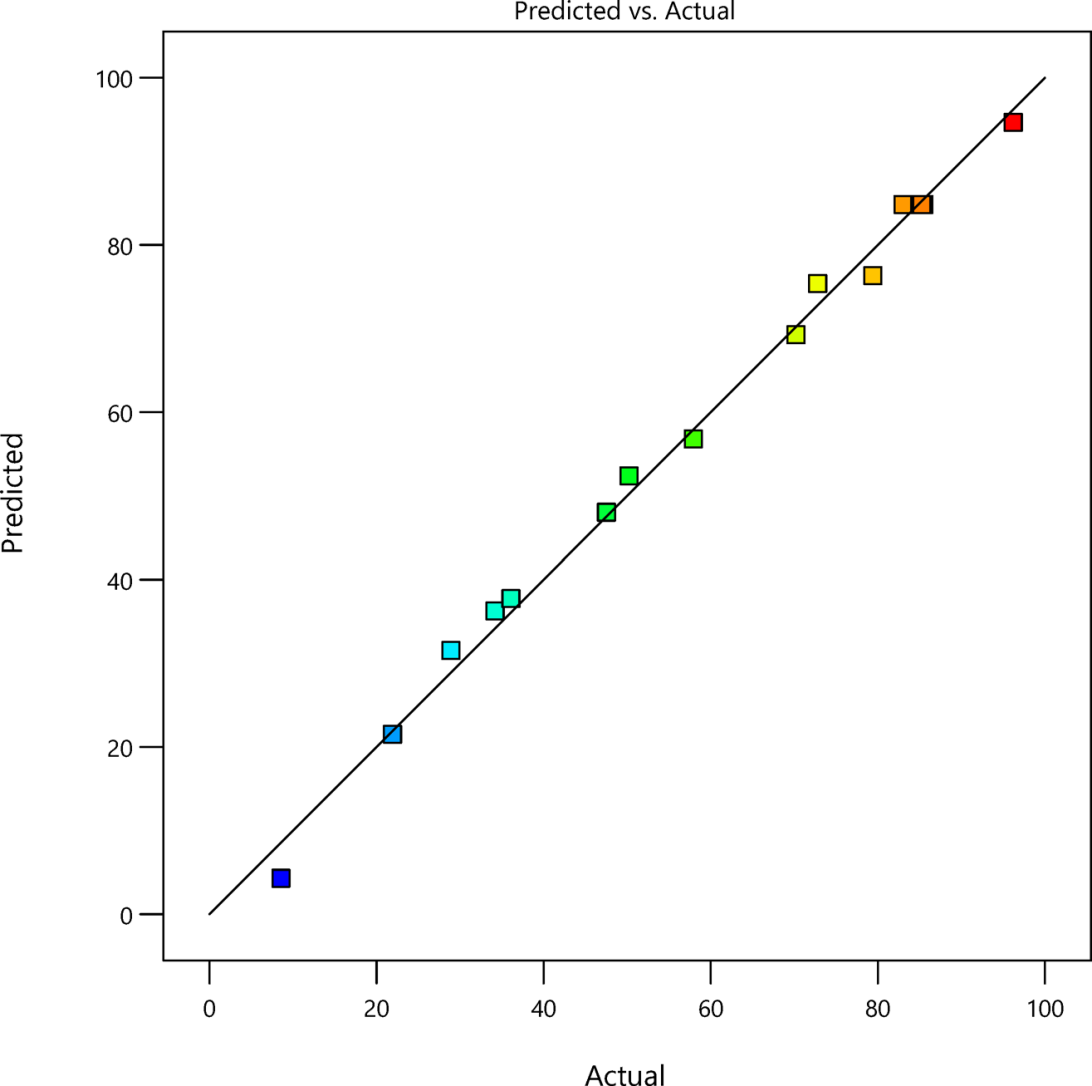
2D plots of actual response vs. predicted response.

Upon evaluating the F-value and analyzing the preceding data, it is obvious that the effect of the factors on the removal percentage was as follows: dosage > pH > concentration. The ANOVA results provided several statistical model coefficients to evaluate the model’s quality, repeatability, and reliability. The coefficients’ values are presented in Table 3. An R^2^ value of 0.9946 signifies a strong correlation between the actual and predicted values, as illustrated in the 2D plots of these values in Fig. 14, which demonstrates the association between the actual response values and the predicted response values. The predicted R^2^ value of 0.9147 corresponds with the adjusted R^2^ value of 0.9877, and the difference between the two values not exceeds 0.2, signifying that the model is both significant and dependable. Furthermore, the standard deviation value of 2.98 reflects the model’s repeatability. The coefficient of variation (C.V.) of 4.93% signifies the model’s suitability, as it is below 10%. The signal-to-noise ratio or adequate precision coefficient signifies that the model possesses sufficient accuracy to differentiate between noise and signal. A value exceeding 4 signifies that the model is reliable^56.^ All the above indicate that the model is significant, reliable, and reproducible.

**Table 3:** The statistical model coefficients.

Std. Dev.	2.98
Mean	60.48
C.V. %	4.93
R²	0.9946
Adjusted R²	0.9877
Predicted R²	0.9147
Adeq Precision	39.499

#### Investigate the impact of variables and their interaction

**Fig. 15:**
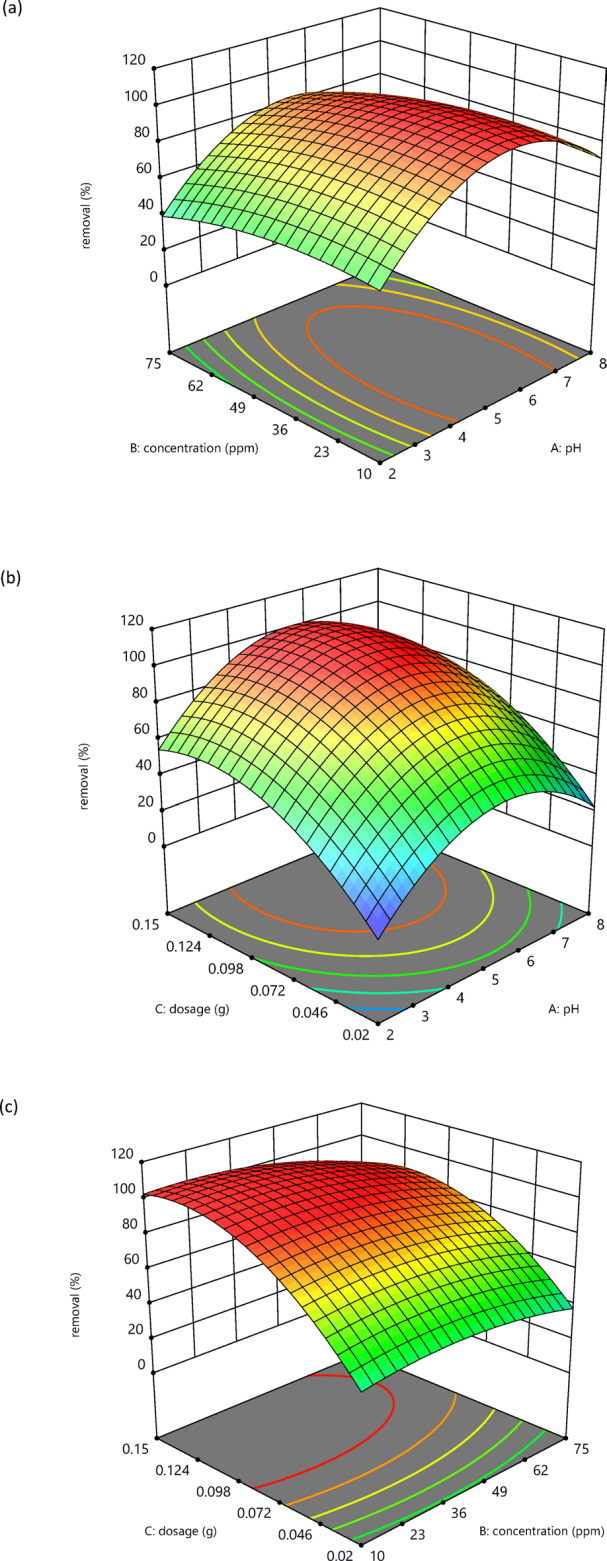
3D plots of interacting variables (**a**: pH vs. concentration, **b**: pH vs. dosage, **c**: concentration vs. dosage) and their effect on response.

The influence of factors on each other and the response designed for BBD-RSM modeling can be studied using 3D graphs. These graphs, as shown in Fig. 15 (a-c), represent the relationship between two variables and their effect on the response, holding the other factor constant. These plots help evaluate the best adsorption conditions to achieve optimal removal performance and understand the interaction effects between variables^57^. It is clear that with increasing dosage of nanoparticles at the same pH and concentration, the removal percentage increases due to the increased number of active sites. The opposite occurs with increasing dye concentration, holding all other factors constant. According to the F-test data, pH is the predominant factor affecting the response; this is due to the dye being anionic and the pH_pzc_ of nanoparticles being 8; thus, electrostatic attraction occurs in acidic conditions, resulting in adsorption and subsequent removal. Consequently, pH is the major factor affecting the response.

### Adsorption kinetics

Kinetics study is crucial in adsorption research since it estimates how a substance is eliminated from water-based solutions. Additionally, it offers vital insights into the underlying mechanisms of sorption events^58^. To examine the mechanism by which Aniline blue dye is adsorbed onto magnesium silicate nanoparticles, pseudo-first order, pseudo-second order, and intra-particle diffusion were utilized. It was found that the non-linear form has an advantage over the linear model because the distribution of the error remains unchanged, and the kinetic factors are fixed on the same ordinates and axis^59^. The optimal model was assessed using R^2^ correlation coefficients, and the $\:{\chi\:}^{2}$ test, with the superior model exhibiting the highest R^2^, and the lowest $\:{\chi\:}^{2}$ value^60^. The following equation gives the non-linear form of the rate equation of pseudo-first order:

4 $$\:{q}_{t}={q}_{e}(1-{e}^{-{K}_{1}t})$$

**Fig. 16:**
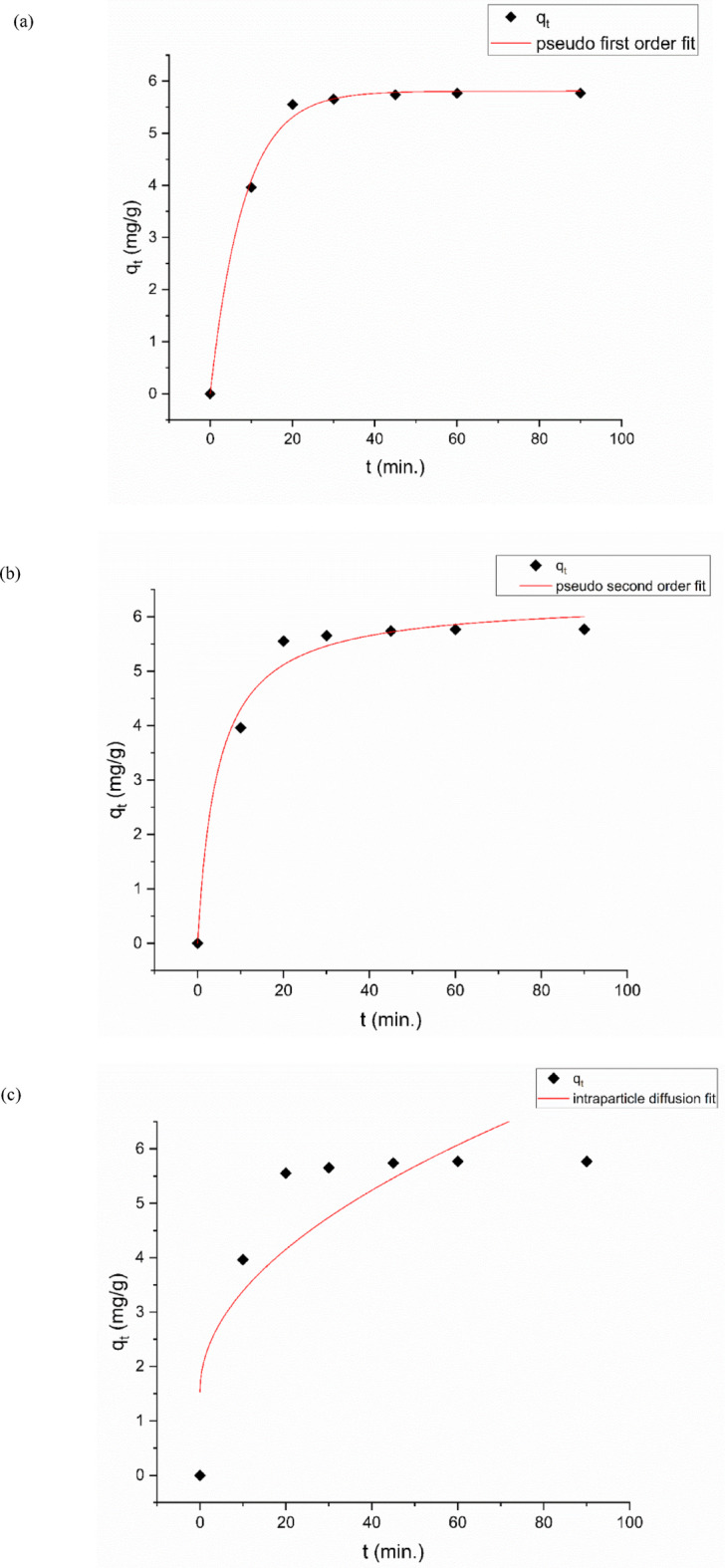
pseudo-first-order (**a**), pseudo-second-order (**b**), intra-particle diffusion (**c**) for the removal of Aniline blue dye onto magnesium silicate nanoparticles.

Where $\:{q}_{e}$ denotes the equilibrium adsorption capacity of the dye, measured in milligrams per gram, $\:{q}_{t}$ Indicates the quantity of dye that has been adsorbed (in milligrams per gram) at a particular time t (in minutes) and $\:{K}_{1}$ ($\:{min}^{-1}$) is the rate constant of the pseudo-first-order. Figure 16 (a) illustrates that by plotting $\:{q}_{t}$ against $\:t$, one can derive $\:{K}_{1}$ and $\:{q}_{e}$.

The rate equation pseudo-second order is shown as Eq. (5):

5 $$\:{q}_{t}=\frac{{q}_{e}^{2}{K}_{2}t}{1+{q}_{e}{K}_{2}t}$$

Where $\:{K}_{2}$ ($\:\frac{g}{mg.min})$ is the pseudo-second-order rate constant, and it may be determined by plotting $\:{q}_{t}$ against $\:t,$ as shown in Fig. 16 (b).

To gain further insight into the mechanism and the potential rate-determining steps of the adsorption process, the intra-particle diffusion model was applied. This model is vital for determining whether dye diffusion within the pores of the adsorbent (intra-particle diffusion) or diffusion across the liquid layer surrounding the particle (film or boundary layer diffusion) controls the adsorption rate. The equation of intra-particle diffusion is shown as:

6 $$\:{q}_{t}={K}_{i}{t}^{0.5}+C$$

Where $\:{K}_{i}$ is the intra-particle diffusion rate constant ($\:\frac{mg}{g.{min}^{0.5}}$) and C is the film thickness. $\:{K}_{i}$ and C values can be obtained after plotting $\:{q}_{t}$ against t, as shown in Fig. 16 (c), the plot exhibits multilinearity, indicating the occurrence of two distinct phases in the adsorption process. The initial step involved the adsorption of molecules on the external surface, while the subsequent step involved a progressive and continuous adsorption process^61^. If the rate-determining step is the intra-particle diffusion, the plot of time (t) versus the amount of substance adsorbed ($\:{q}_{t}$) should show a linear relationship in the linear form and have an intercept (C) equal to zero. The rate-determining step will be controlled by boundary layer (film) diffusion if there is any divergence from linearity^58^.

Table 4 Illustrates that the pseudo-first-order model fitted with the elimination of aniline blue dye from aquatic solutions using magnesium silicate nanoparticles due to $\:{R}^{2}$ is more than 0.99, $\:{\chi\:}^{2}$ = 0.017, and the value of $\:{q}_{{e}_{\left(Theoretical\right)}}$ is 5.81 ± 0.07 mg/g, which is close to $\:{q}_{{e}_{\left(experimental\right)}}$, and it is observed that the pseudo-second-order is not suited because of $\:{R}^{2}$ is less than 0.99, $\:{\chi\:}^{2}$ = 0.081, and $\:{q}_{{e}_{\left(Theoretical\right)}}$ is 6.13 ± 0.25 mg/g. The fact that neither linear region passes through the origin (C ≠ 0) confirms that intra-particle diffusion is not the sole rate-limiting mechanism and that film diffusion also plays a significant role in controlling the adsorption kinetics.

**Table 4:** The kinetic parameters for the adsorption of aniline blue dye on magnesium silicate nanoparticles.

Kinetic model	Parameters	Results
pseudo-first order	$\:{q}_{{e}_{\left(experimental\right)}}$ mg/g	5.77
	$\:{q}_{{e}_{\left(Theoretical\right)}}$ mg/g	5.81 ± 0.07
	$\:{K}_{1}$ ($\:{min}^{-1}$)	0.12 ± 0.01
	$\:{R}^{2}$	0.996
	$\:{\chi\:}^{2}$	0.017
pseudo-second order	$\:{q}_{{e}_{\left(experimental\right)}}$ mg/g	5.77
	$\:{q}_{{e}_{\left(Theoretical\right)}}$ mg/g	6.13 ± 0.25
	$\:{K}_{2}$ ($\:\frac{g}{mg.min}$)	0.03 ± 0.01
	$\:{R}^{2}$	0.985
	$\:{\chi\:}^{2}$	0.081
intra-particle diffusion	C	1.53 ± 0.96
	$\:{K}_{i}$ ($\:\frac{mg}{g.{min}^{0.5}}$)	0.59 ± 0.16
	$\:{R}^{2}$	0.732
	$\:{\chi\:}^{2}$	1.478

### Adsorption isotherm

The adsorption isotherm is essential for elucidating the fundamental concepts that regulate the accumulation, discharge, or migration of chemicals from water-based porous materials or aquatic environments to a solid phase while maintaining constant temperature and pH^62^. Non-linear regression analysis was employed to estimate diverse isotherm parameters and determine the optimal correlation to elucidate experimental data. Non-linear isotherm modeling is better for estimating isotherm parameters. This is because linearization results in inherent bias, diverse estimation errors, and fit distortions^60^.

#### Langmuir isotherm

This model supposes that adsorption occurs only on a single layer, known as a monolayer, with a uniform surface on the adsorbent. In this model, the adsorption energy remains consistent throughout all active sites, and interaction is absent among the adsorbent molecules owing to the immobilization of the adsorbate on the outer layer of the adsorbent^63.^ Eq. (7) represents the Langmuir equation in its non-linear form:

7 $$\:{q}_{e}=\frac{{q}_{o}b{C}_{e}}{1+b{C}_{e}}$$

**Fig. 17:**
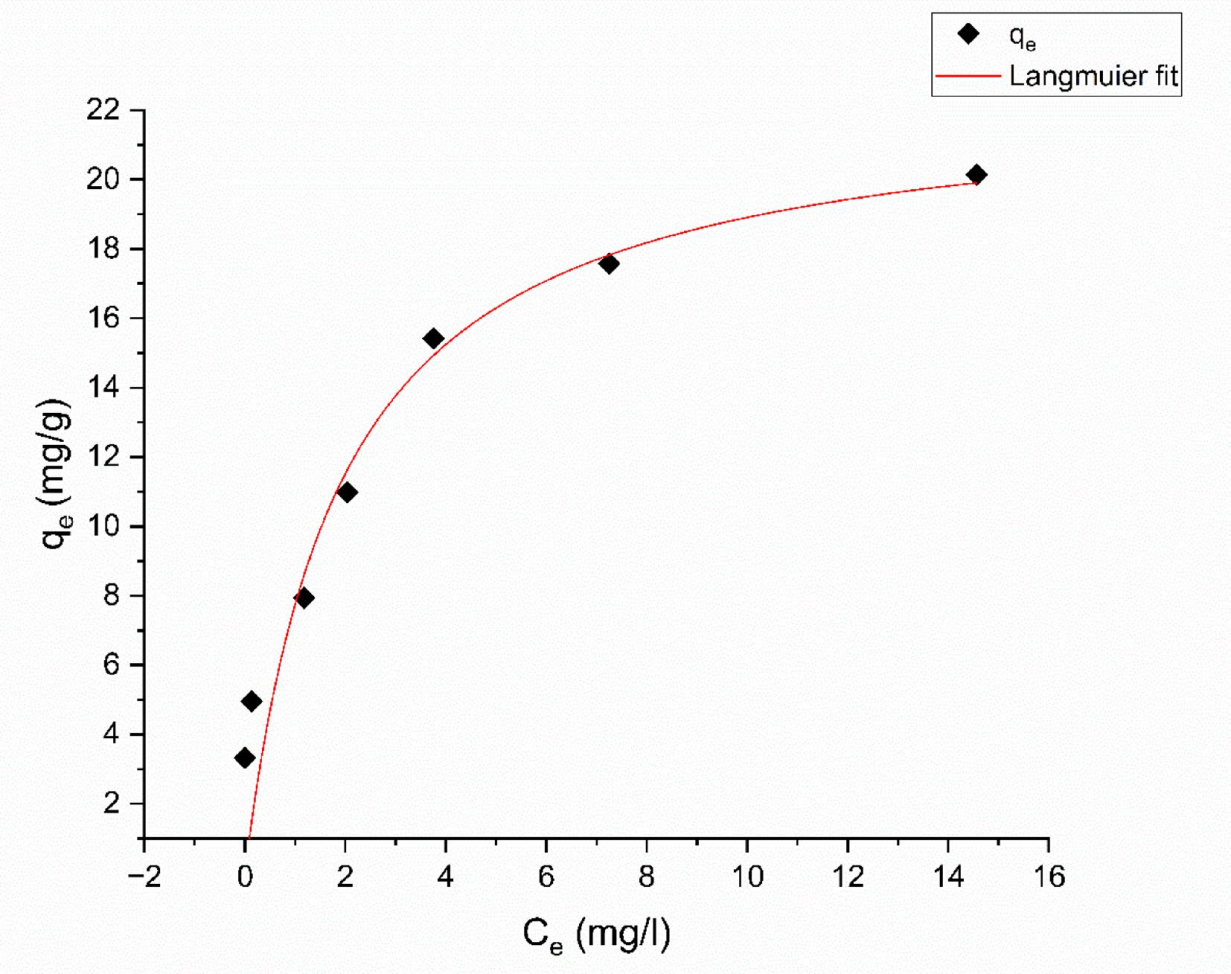
Langmuir adsorption isotherm of Aniline blue dye onto magnesium silicate nanoparticles.

In this context, $\:{q}_{e}$ (mg/g) represents the quantity of dye per unit mass of adsorbent at equilibrium, $\:{q}_{o}$ (mg/g) represents the maximal adsorption capacity, C_e_ (mg/L) represents the concentration of dye at equilibrium, and the Langmuir constant, b (L/mg), is a measure of the energy of adsorption. It indicates the propensity of the adsorbate to bind to the sites that are actively present on the surface of the adsorbent. An increased value of b signifies enhanced adsorption energy. Langmuir isotherm parameters $\:{q}_{o}$ and b were determined, when $\:{q}_{e}$ is plotted against $\:{C}_{e}$ as shown in Fig. 17.

Langmuir isotherm parameters are shown in Table 5. The correlation coefficient value ($\:{R}^{2}$ $\:>0.90$), and $\:{\chi\:}^{2}$ = 4.828 showed that this model is not fitted for the adsorption of Aniline blue dye onto magnesium silicate nanoparticles. The highest adsorption capacity ($\:{q}_{o}$) was 22.52 mg/g. The separation factor ($\:{R}_{L}$) is utilized for predicting the adsorption effectiveness, and the applicability of the Langmuir equation is determined by Eq. (8):

8 $$\:{R}_{L}=\frac{1}{1+b{C}_{o}}$$

Where b (L/mg) is the Langmuir constant, and $\:{C}_{o}$(mg/L) is the initial concentration. When the $\:{R}_{L}$value ranges from 0 to 1, which implies favorable adsorption. A $\:{R}_{L}$value greater than 1 suggests unfavorable adsorption, while a $\:{R}_{L}$value of 0 indicates irreversible adsorption. A $\:{R}_{L}$value of 1 signifies linear adsorption. Table 5 displays the values of $\:{R}_{L}$ at various dye concentrations, indicating favorable adsorption.

**Table 5:** Values of the separation factor (R_L_).

$\:{C}_{o}$ (ppm)	$\:{R}_{L}$
10	0.16
15	0.11
25	0.07
35	0.05
50	0.04
60	0.03
75	0.03

#### Freundlich isotherm

The Freundlich isotherm model is an empirical equation that provides an alternative to the Langmuir model for analyzing multiple-layer adsorption. This model proposes that the outer layer of the adsorbent is not uniform but consists of active sites with varying energies dispersed exponentially. Initially, the more powerful binding sites are filled, leading to a steady reduction in adsorption energy as the adsorption process nears its end^63^. The equation that delineates the non-linear representation of the Freundlich isotherm model is articulated as follows:

9 $$\:{q}_{e}={K}_{f}{C}_{e}^{\frac{1}{{n}_{f}}}$$

**Fig. 18:**
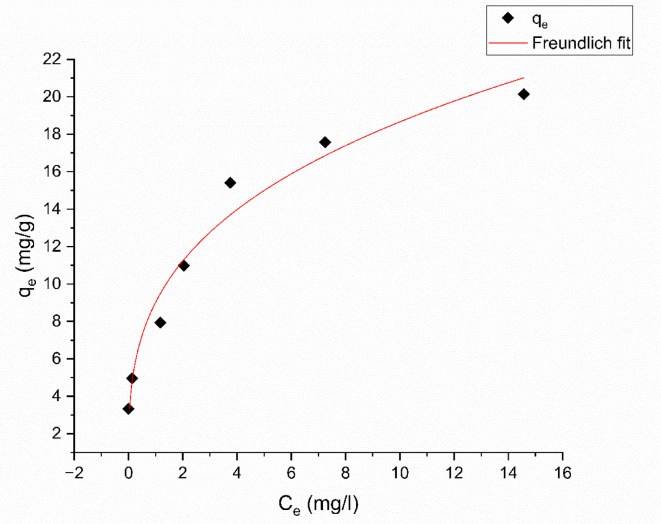
Freundlich adsorption isotherm of Aniline blue dye onto magnesium silicate nanoparticles.

Where the adsorption coefficient, denoted as $\:{K}_{f}$, assesses the adhesive ability of the adsorbate to the adsorbent, reflecting the relative adsorption potential of the adsorbent, $\:\frac{1}{n}$ denotes the degree of adsorption of an adsorbate onto an adsorbent or the variability of a surface, $\:{q}_{e}$, and C_e_ as denoted in the Langmuir isotherm. Freundlich isotherm parameters ($\:{K}_{f}$ and n) may be acquired after plotting $\:{q}_{e}\:$versus$\:\:{C}_{e}$, as shown in Fig. 18.

The Freundlich isotherm parameters are shown in Table 6. The parameter n measures the extent to which adsorption is favorable. When the n-values range from 1 to 10, the adsorption isotherm is regarded as favorable. Conversely, an adsorption isotherm with *n* < 1 is deemed unfavorable. As shown in Table 6, the value of n is 3.16; therefore, it suggests that the adsorption is favorable. Furthermore, as the value of $\:{K}_{f}$ increases, so does the adsorption capacity of the adsorbent. This model shows high regression coefficient ($\:{R}^{2}$ > 0.96), and $\:{\chi\:}^{2}$ = 1.683, which is the lowest value, indicating that this model was fitted with the adsorption of aniline blue dye onto magnesium silicate nanoparticles.

#### Temkin isotherm

The Temkin isotherm is often used to describe the nonuniform sorption heat distribution^64^. The Temkin isotherm model considers the interactions between the adsorbate and adsorbent, positing a linear decrease in the heat of adsorption, in contrast to the logarithmic decline suggested by the Freundlich equation^58^. Equation (10) represents the non-linear form of the Temkin isotherm:

10 $$\:{q}_{e}=Bln\left(A{C}_{e}\right)$$

Where

11 $$\:B=\frac{RT}{b}$$

**Fig. 19:**
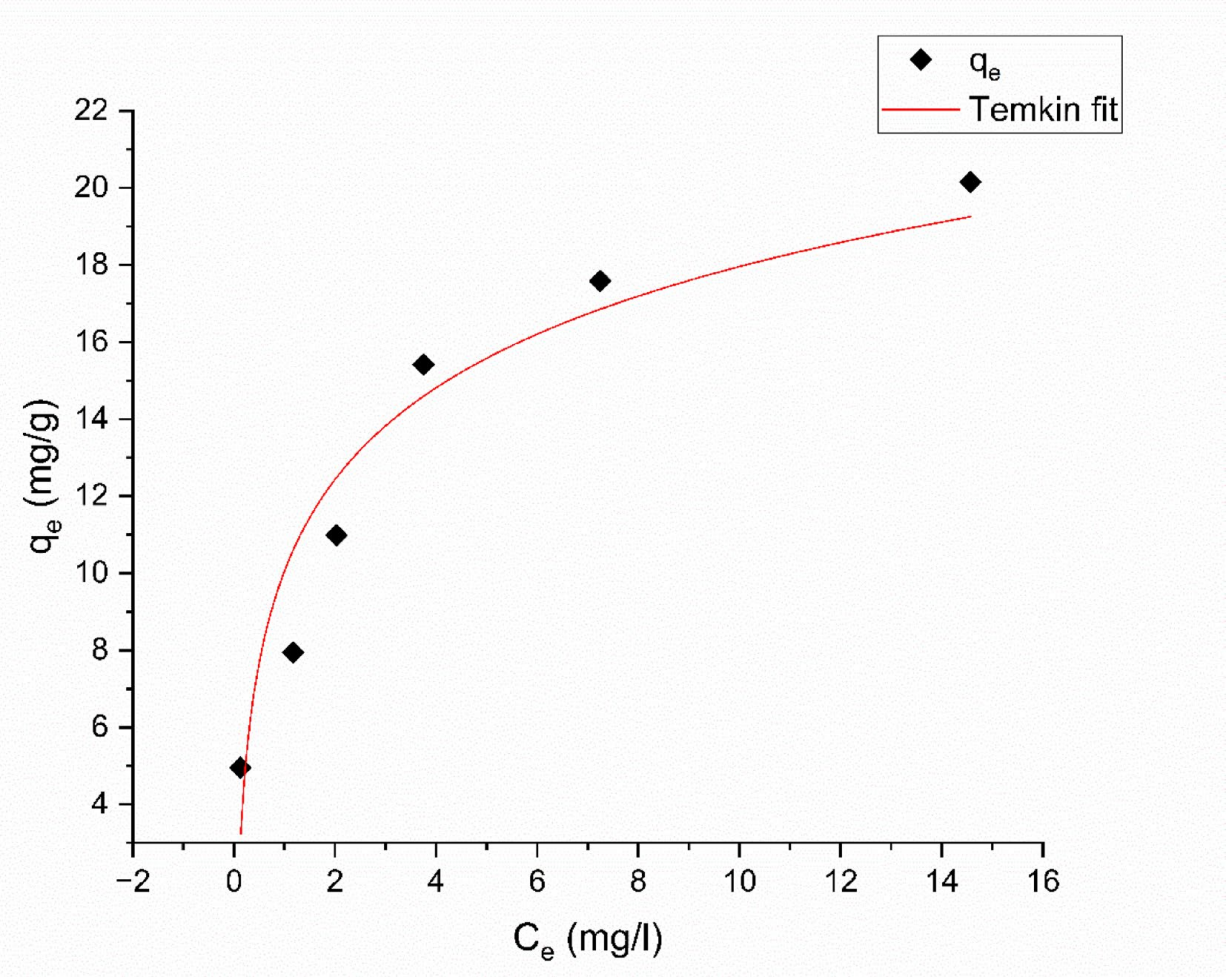
Temkin adsorption isotherm of Aniline blue dye onto magnesium silicate nanoparticles.

A (L/mol) is the equilibrium binding constant representing the maximum binding energy level. B is the heat constant of adsorption, R is the universal gas constant (8.314 J.*mol*^-1^.K^− 1^), T is the temperature (298 K), and b (J/mol) is the Temkin isotherm constant. As shown in Fig. 19, when $\:{q}_{e}$ is plotting against $\:{C}_{e}$, the Temkin constants (B and A) may be derived after plotting the data. Temkin isotherm parameters are provided in Table 6.

#### The Dubinin-Kaganer-Radushkevick (DKR)

This isotherm represents a mathematical model offering a more detailed approach than the Langmuir isotherm, as it does not assume a uniform surface or a constant potential for adsorption^19^.

It describes the adsorption of Aniline blue dye on mesoporous substances. It specifically focuses on the process of pore-filling. This method differentiates between physical and chemical adsorption, allowing a molecule to release from its position in the sorption space to an unrestricted distance^65^. The non-linear form of this isotherm is used to calculate the apparent energy of Aniline blue dye adsorption onto magnesium silicate nanoparticles^64^. This model is represented by Eq. (12):

12 $$\:{q}_{e}={q}_{max}{e}^{-\beta\:{\left[RTln\left(1+\frac{1}{{C}_{e}}\right)\right]}^{2}}$$

**Fig. 20:**
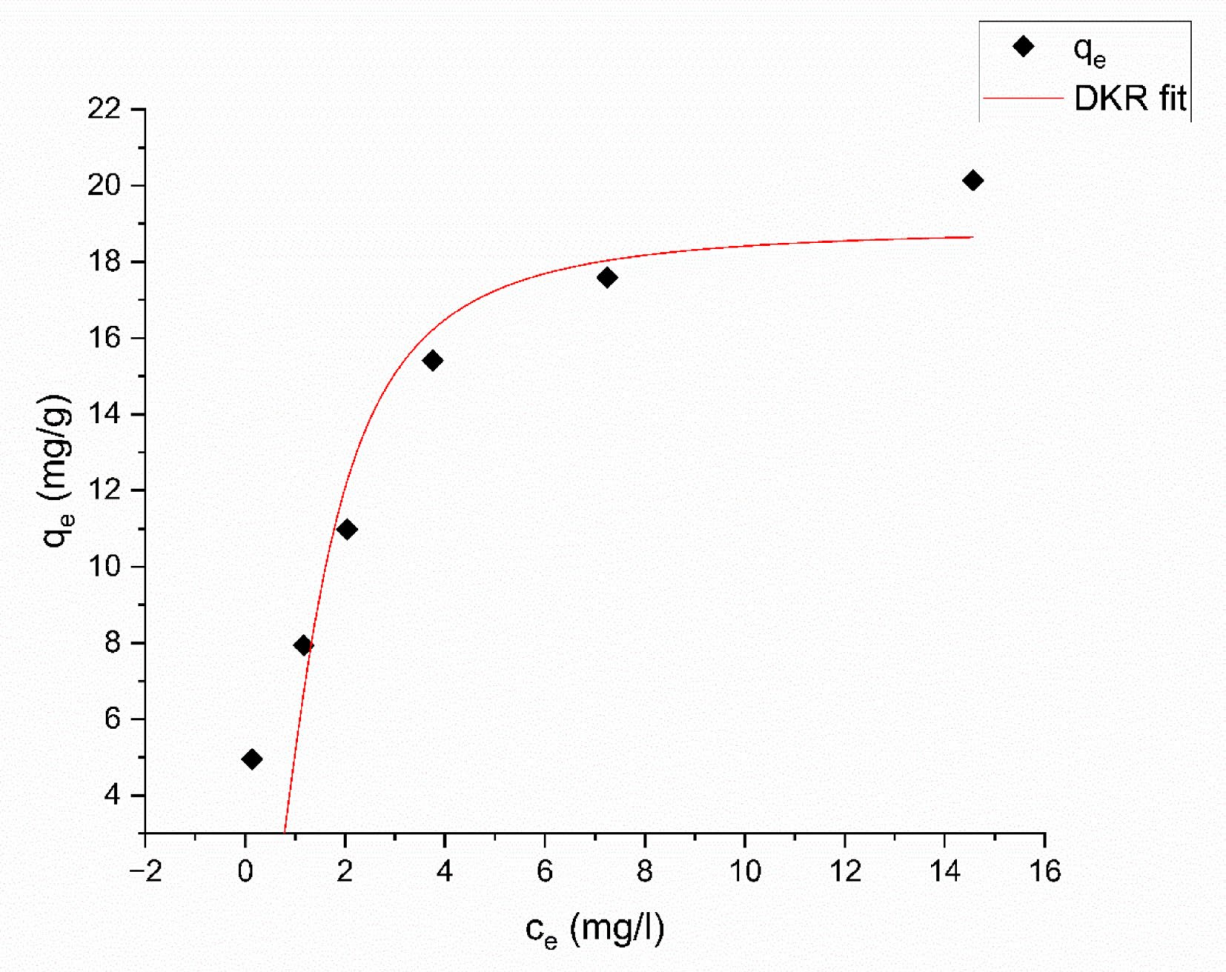
DKR adsorption isotherm of Aniline blue dye onto magnesium silicate nanoparticles.

Where $\:{q}_{max}$stands for maximal sorption capacity (mg/g), $\:{\left(\frac{mol}{kJ}\right)}^{2}$for an activity coefficient constant relating to sorption energy, C_e_ (mg/L) represents the concentration of dye at equilibrium, R is the universal gas constant (8.314 J.*mol*^−1^.K^− 1^), and T is temperature (298 K). When $\:{q}_{e}$ plotted against $\:{C}_{e}$, as shown in Fig. 20, $\:{q}_{max}$ and $\:\beta\:$ can be obtained.

The constant, β, is the average amount of energy required for each mole of sorbate to migrate from an infinite distance in the solution to the outer layer of the solid^63^. The energy may be determined using Eq. (13):

13 $$\:E=\frac{1}{\sqrt{2\beta\:}}$$

The adsorption process is mainly determined by the mean free energy (E) value. If the energy (E) falls between the range of 8 to 16 $\:\frac{kJ}{mol}$, the adsorption process may be classified as ion exchange. On the other hand, if the magnitude of *E* exceeds 16 $\:\frac{kJ}{mol}$ then, the adsorption process is categorized as a chemical interaction. If the energy is less than 8 $\:\frac{kJ}{mol}$, the adsorption process is categorized as physical contact. As shown in Table 6, the value of E is 4.29 × 10^−4^ $\:\frac{kJ}{mol}$, according to this value, the process of adsorption is a physical interaction.

According to $\:{R}^{2}$ ,and $\:{\chi\:}^{2}$ results, the Freundlich isotherm model is more suited for the adsorption of Aniline blue dye onto magnesium silicate nanoparticles.

**Table 6:** Values of langmuir, freundlich, DKR, TEMKIN, and R^2^ for the adsorption of aniline blue dye on nano magnesium silicate nanoparticles.

Isotherm	Results
Langmuir isotherm	$\:{q}_{max}$= 22.52 ± 2.86 mg/g b = 0.52 ± 0.22 L/mg R^2^ = 0.90 $\:{\chi\:}^{2}$ = 4.828
Freundlich isotherm	K_f_ = 9.02 ± 0.71 (mg g^− 1^) (L g^− 1^)^1/*n*^ *n* = 3.17 ± 0.40 1/*n* = 0.32 ± 0.04 R^2^ = 0.96 $\:{\chi\:}^{2}$ = 1.683
Temkin isotherm	B = 3.43 ± 0.52 b = 722.32 ± 95.10 J mol^− 1^ A = 18.7 ± 11.38 L/mol R^2^ = 0.916 $\:{\chi\:}^{2}$ = 3.582
DKR isotherm	E = 4.29 × 10^− 4^ ± 0.86 × 10^− 4^ kJ mol^− 1^ q_max_ = 18.87 ± 1.87 mg/g R^2^ = 0.82 $\:{\chi\:}^{2}$ = 7.661

### Adsorption thermodynamics

**Fig. 21:**
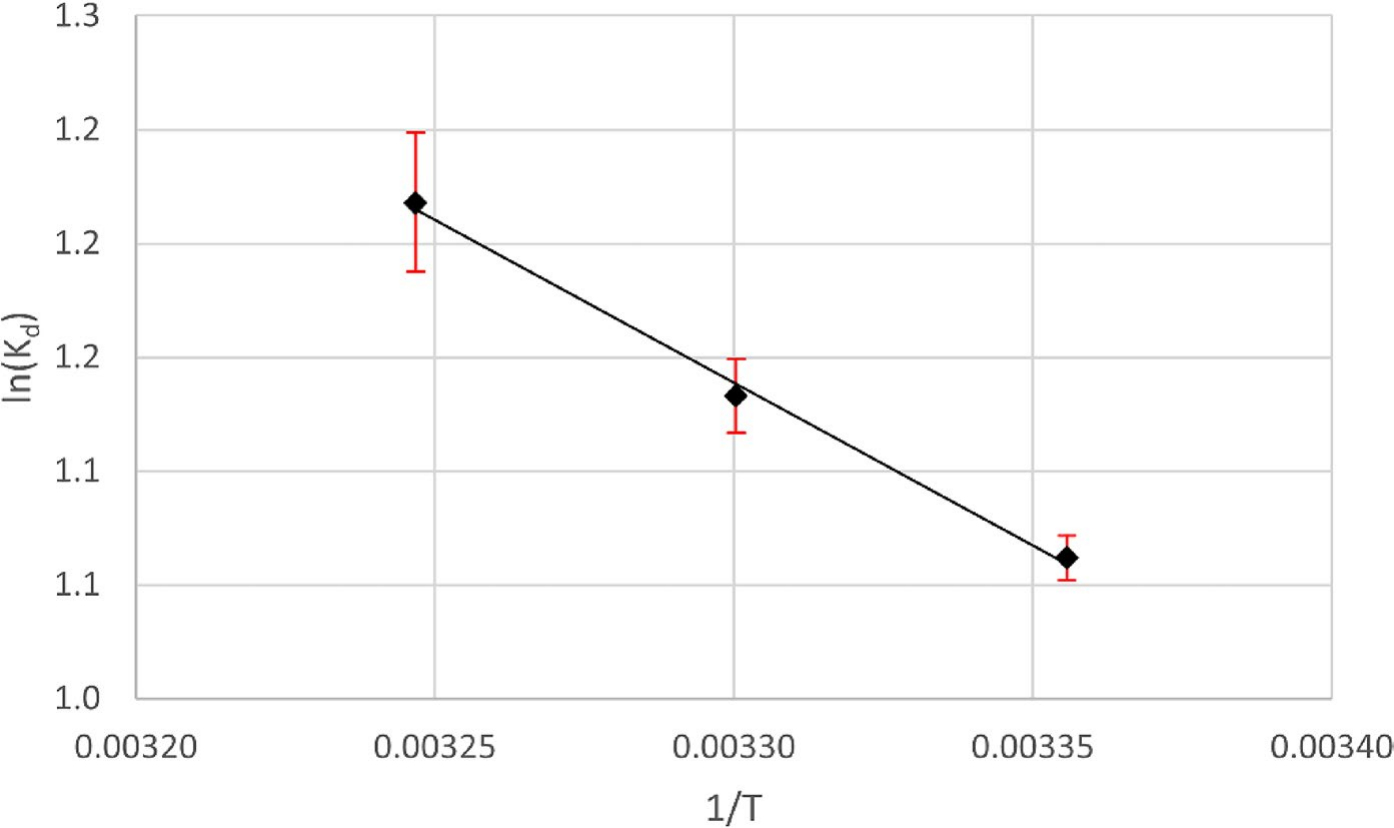
Van’t Hoff plot of aniline blue dye adsorbed onto magnesium silicate nanoparticles.

Thermodynamic parameters are utilized to assess the spontaneity of an adsorption process. The changes in free energy (ΔG°), enthalpy (ΔH°), and entropy (ΔS°) of the adsorption process were determined using the following equations:

14 $$\:{K}_{d}=\frac{{q}_{e}}{{C}_{e}}$$

15 $$\:\varDelta\:{G}^{o}=-RTln{K}_{d}$$

16 $$\:ln{K}_{d}=\frac{-(\varDelta\:{H}^{o})}{RT}+\frac{\varDelta\:{S}^{o}}{R}$$

q_e_ represents the equilibrium concentration of adsorbed dye on the adsorbent, while C_e_ represents the equilibrium concentration of the residual dye in the solution. A linear relationship is observed in a Van’t Hoff plot of the ln k_d_ vs. the reciprocal of temperature (1/T)) Fig. 21).

The values of ΔH° and ΔS° can be calculated by calculating the slope and intercept of the line. The ΔH° value of 9.53 kJ mol^− 1^ demonstrates that the adsorption of aniline blue onto magnesium silicate nanoparticles is an endothermic process. The positive ΔS° value of 0.041 kJ mol^− 1^K^− 1^ indicates an increase in randomness at the adsorption/solution interface during the adsorption of the dye onto nano magnesium silicate. The ΔG° values at different temperatures 298, 303, and 308 K were − 2.69, -2.88, and − 3.11 kJ mol^− 1^, respectively, which indicate that aniline blue adsorption is a spontaneous process^66^.

### Application in real samples

**Fig. 22:**
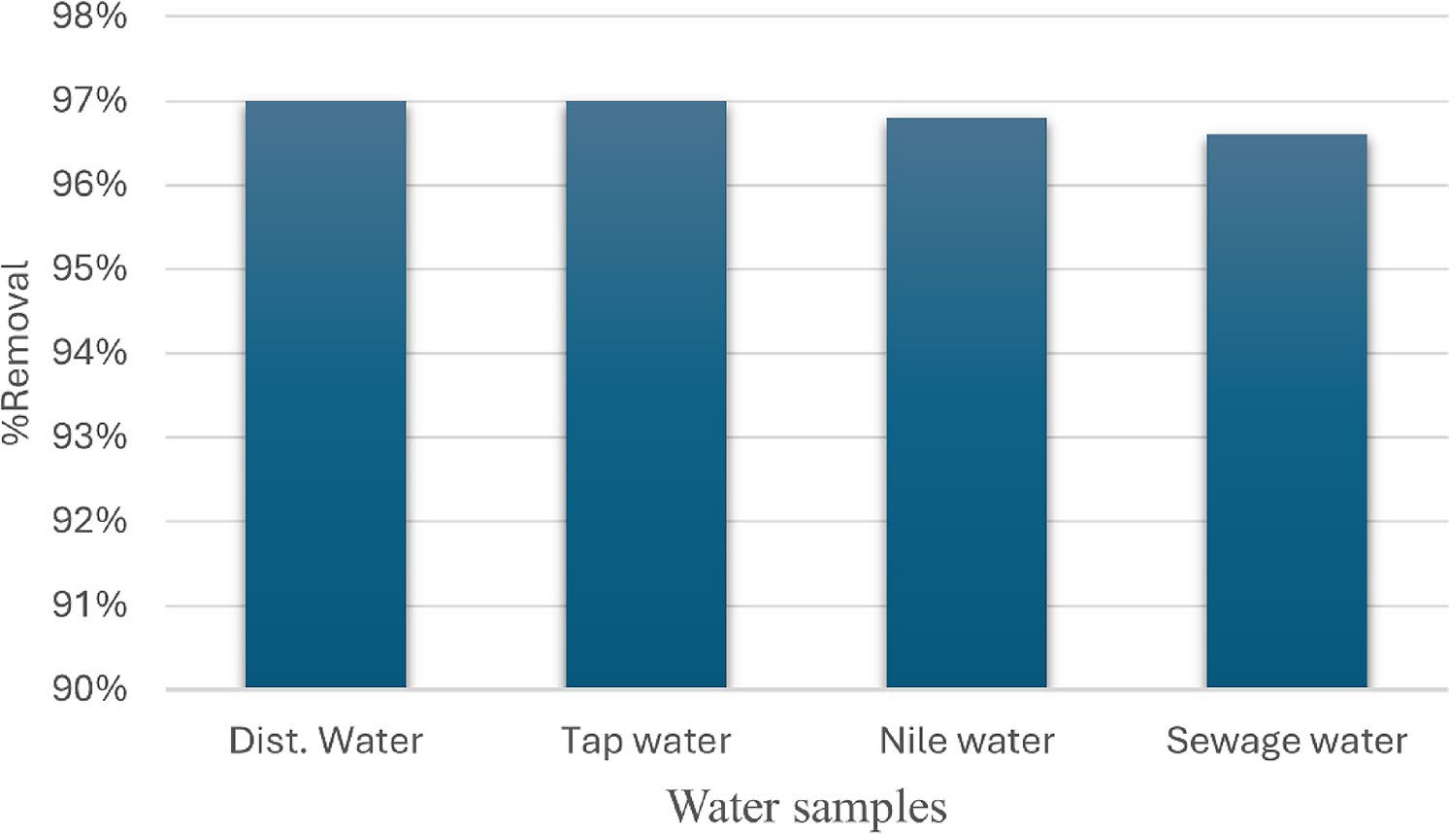
Removal percentage of aniline blue dye from various real samples.

The interactions between magnesium silicate nanoparticles and aniline blue dye under ideal conditions were studied using various water sources, including sewage water, tap water, and Nile water (Fig. 22). We have prepared a reference solution containing 10 ppm of aniline blue dye to assist with our analysis. Samples for laboratory testing were gathered from tap water, a nearby section of the Nile, and an Egyptian sewage treatment facility plant. All samples were filtered through 0.45 μm filters prior to the adsorption tests in order to remove any suspended particles. Notable results were obtained from our evaluation of the performance of magnesium silicate nanoparticles in these real water samples, as shown in Fig. 22. Magnesium silicate nanoparticles demonstrated a remarkable dye removal efficiency, underscoring their utility. The effectiveness of magnesium silicate nanoparticles in eliminating dyes from wastewater has been demonstrated. This study demonstrates how magnesium silicate nanoparticles may be used in practical applications as an efficient dye removal method in a variety of water sources.

### Regeneration

**Fig. 23:**
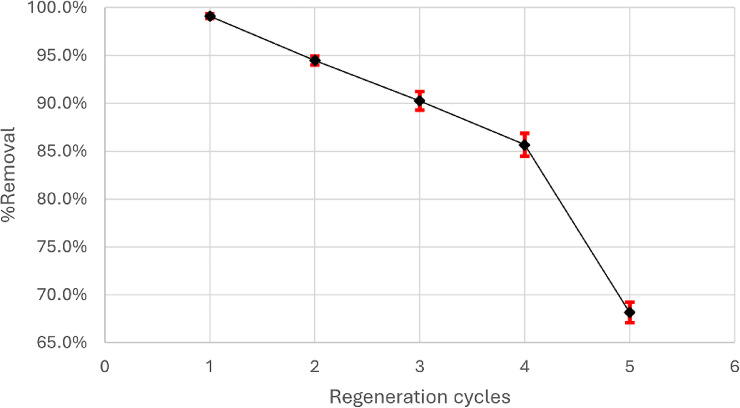
Regeneration cycles of magnesium silicate nanoparticles.

The regeneration procedure is crucial for evaluating the sustainability and cost-effectiveness of magnesium silicate. Magnesium silicate nanoparticles were washed with 0.01 M HCl and NaOH after each cycle to enable regeneration and were subsequently rinsed with bi-distilled water. Magnesium silicate nanoparticles are employed in five adsorption and desorption cycles, as demonstrated in Fig. 23. Following cycle no. 4, the removal percentage dropped to 68% due to the reduced number of active sites attributed to adsorption-desorption processes.

### Comparison with previous studies

As shown in Table 7, magnesium silicate nanoparticles can remove aniline blue dye at a high rate, with a smaller quantity of nanoparticles, and in a short time compared to some other materials that have been used to remove the same dye.

**Table 7:** Comparison between different types of adsorbents for the removal of aniline blue dye.

Adsorbent	Treatment	Adsorption kinetics	Adsorption isotherm	Contact time (minutes)	Initial concentration (ppm)	Dosage (g/l)	Removal (%)	Reference
acidified palygorskite/BiOI composites	adsorption-photoactivity	second order	Freundlich	360	50	2	90	^26^
iodo polyurethane foam	adsorption	second order	Freundlich	30	18	4	80–100	^67^
activated carbon coated by chitosan	adsorption	second order	Freundlich	100	50	4	80	^27^
magnesium silicate	adsorption	first order	Freundlich	30	50	3	90	this work

The maximum adsorption capacity (q_e_) of the synthesized magnesium silicate nanoparticles for aniline blue dye was found to be 21 mg/g. This value was compared with previously reported adsorbents, as summarized in Table 8. While two adsorbents, polyvinyl alcohol assisted iron–zinc nanocomposite (92.59 mg/g) and polypyrrole-decorated bentonite magnetic nanocomposite (78.74 mg/g) exhibited slightly higher q_e_ values, the current material still shows competitive performance. Furthermore, the other two materials, neem sawdust (4.354 mg/g) and neem leaf powder (8.76 mg/g), demonstrated lower adsorption capacities than the present study. It is worth noting that, in addition to its comparable adsorption capacity, the synthesized magnesium silicate offers advantages such as fast adsorption rate, low cost, ease of synthesis, environmental friendliness, and high stability, which support its potential for practical applications in dye removal.

**Table 8:** Comparison between values of maximum sorption capacity for different adsorbents.

Dye	Adsorbent	Maximum sorption capacity (mg/g)	Reference
malachite green	polyvinyl alcohol assisted iron–zinc nanocomposite	92.59	^68^
crystal violet	polypyrrole-decorated bentonite magnetic nanocomposite	78.74	^69^
malachite green	neem sawdust	4.354	^70^
methylene blue	neem leaf powder	8.76	^71^
aniline blue	magnesium silicate	22.52	this work

## Conclusion

The magnesium silicate nanoparticles were prepared using the sol-gel method, and their characteristics were assessed using various techniques such as transmission electron microscopy (TEM), X-ray diffraction (XRD), Brunauer-Emmett-Teller (BET) analysis, and fourier transform infrared spectroscopy (FTIR). The anionic dye, aniline blue, was effectively eliminated from the aqueous solution under ambient conditions (room temperature) and pH 4 with a dosage of 3 g/l of nanoparticles, and the agitation speed was adjusted to 100 revolutions per minute (rpm). The percentage of elimination was 99% within the initial 30 min. The adsorption process adhered to the Freundlich isotherm with a correlation coefficient ($\:{R}^{2}$) of 0.96, and it followed the pseudo-first-order kinetics with a correlation coefficient ($\:{R}^{2}$) of 0.996. Additionally, it was determined that the process of elimination involved physisorption, and the rate-determining step (RDS) was the boundary layer (film) diffusion. From the study of thermodynamics, the process was spontaneous and endothermic.

## Supplementary Information

Below is the link to the electronic supplementary material.

Supplementary Material 1

## Data Availability

All data generated or analyzed during this study are included in this published article [and its supplementary information files].
