# Supplementary material for: Integrated kinetic, thermodynamic, and statistical investigation of aniline blue dye removal using magnesium silicate nanoparticles
Source: Sci Rep. 2025 Sep 24;15:32691. doi: 10.1038/s41598-025-18726-z (PMC12460873; doi:10.1038/s41598-025-18726-z)
Supplement: Supplementary file 1 — Supplementary Material 1 [file 41598_2025_18726_MOESM1_ESM.docx]

## **Integrated Kinetic, Thermodynamic, and Statistical Investigation of Aniline Blue Dye Removal Using Magnesium Silicate Nanoparticles**

## ***Ahmed Magdy*** ******^1^, Magdi E.A. Zaki*********^2^, Maysa R. Mostafa^1^, Gehad G. Mohamed^1,3^,*** ***Sami A. Al-Hussain^2^, Omar A. Fouad*** ******^1^***

1. Chemistry Department, Faculty of Science, Cairo University, 12613, Giza, Egypt.
2. Department of Chemistry, Faculty of Science, Imam Mohammad Ibn Saud Islamic University (IMSIU), Riyadh 11623, Saudi Arabia
3. Nanoscience Department, Basic and Applied Sciences Institute, Egypt-Japan University of Science and Technology, New Borg El Arab, Alexandria, 21934, Egypt

*** Ahmed Magdy: The corresponding author**

**E-mail: ahmed.magdy199880@gmail.com**

*** Omar A. Fouad: The corresponding author**

E-mail: [oahmed@sci.cu.edu.eg](mailto:oahmed@sci.cu.edu.eg)

*** ***Magdi E.A. Zaki:* The corresponding author**

**E-mail:** [mezaki@imamu.edu.sa](mailto:mezaki@imamu.edu.sa)

## **Apparatus**

The Egypt Nanotechnology Center (EGNC) used the Bruker D8 Discover (Bruker AXS Inc., 35 KV, 30 mA) X-ray diffractometer to record the X-ray diffraction (XRD) using Cu Kα radiation (λ = 1.5406 Å) for two hours with 2θ changing from five to fifty. The speed scan was 0.016 and the step size was 0.02. To find the BET surface area and pore size distribution, gas adsorption tests have been carried out at 77 K using N_2_ as the adsorptive gas. The materials underwent a four-to-twelve-hour high vacuum evacuation before the adsorption trials. Based on the Brunauer-Emmett-Teller (BET) theory, the computation was done, and the analysis was carried out using a Nova Touch LX2 analyzer. To evaluate the pH, the Ag/AgCl double-junction reference electrode (HANNA, HI 5311) included with the Model (Hanna, model 8417) was utilized. All glassware tubes with a conical bottom were cleaned with deionized water and then dried.

**Reagents and Chemicals**

All materials of analytical grade and used as purchased. Sodium hydroxide (NaOH) pellets were purchased from (Honeywell-Germany), magnesium chloride hexahydrate (purity 99%, Sigma-Aldrich Chemie GmbH), tetraethyl orthosilicate TEOS (purity 98%, Sigma-Aldrich Chemie GmbH), ethanol (purity 95%, Sigma-Aldrich Chemie GmbH), and ammonia solution (NH_4_OH Riedel-deHaen, Germany), the commercially available water-soluble aniline blue (triphenylmethane dye that has three sulfonic acid groups), anionic dye, which empirical formula C_32_H_25_N_3_O_9_S_3_Na_2_ and molecular weight is 737.73 g/mol, and maximum absorption at ƛ = 597 nm, obtained from Sigma-Aldrich and used as received without any further purification. Stock solution of aniline blue dye was prepared in distilled water. The desired concentrations were obtained by dilution. The concentrations of the studied dye were measured using the spectrophotometric method. The calibration curve of the dye was prepared by measuring its absorbance against concentration at ƛ_max_ = 597 nm using a UV-Vis spectrophotometer. The final dye concentration was determined spectrophotometrically corresponding to λ_max_ of the dye using the Beer-Lambert equation.
